# Effects of constant and diel cyclic temperatures on the liver and intestinal phospholipid fatty acid composition in rainbow trout *Oncorhynchus mykiss* during seawater acclimation

**DOI:** 10.1186/s40850-021-00086-6

**Published:** 2021-06-21

**Authors:** Jian Ge, Yangen Zhou, Ming Huang, Qinfeng Gao, Yunwei Dong, Shuanglin Dong

**Affiliations:** 1 Key Laboratory of Mariculture, Ministry of Education, Ocean University of China, Qingdao, 266100 Shandong Province China; 2 Function Laboratory for Marine Fisheries Science and Food Production Processes, Qingdao National Laboratory for Marine Science and Technology, Qingdao, 266235 Shandong Province China

**Keywords:** Constant temperature, Diel cyclic temperature, Phospholipid fatty acid composition, Rainbow trout, Seawater acclimation

## Abstract

**Background:**

Rainbow trout is an economically important fish in aquaculture and is a model species in environmental physiology. Despite earlier research on the seawater adaptability of rainbow trout at different temperature regimes, the influence on the liver and intestine in this species is still unknown. Two trials were conducted to investigate the effects of constant and diel cyclic temperatures on phospholipid fatty acid (PLFA) composition in the liver and intestine of rainbow trout during seawater acclimation.

**Results:**

At the end of growth trial 1, fish at 9 and 12.5 °C showed significantly higher ratios of unsaturated to saturated (U/S) and unsaturation index (UI) than those at 16 °C in liver and intestine phospholipids. After day 1 of seawater acclimation, the U/S, UI, and average chain length (ACL) of liver and intestinal phospholipids in fish at 16 °C significantly increased. Two weeks after seawater acclimation, the liver and intestinal PLFA composition adapted to salinity changes. In trial 2, significantly higher U/S, UI, and ACL were found in intestinal phospholipids at 13 ± 2 °C. On the first day after seawater acclimation, UI and ACL in liver phospholipids significantly increased at 13 °C, while fish at 13 ± 2 °C showed significantly decreased U/S, UI, and ACL in the intestine. At the end of growth trial 2, liver PLFA compositions were stable, whereas intestinal PLFA at 13 and 13 ± 1 °C showed significantly decreased U/S, UI, and ACL. A two-way analysis of variance and principal component analysis revealed significant effects of different constant temperatures, seawater acclimation, and their interaction on the liver and intestinal phospholipids, a significant effect of diel cyclic temperature on intestinal phospholipids, and the effects of seawater acclimation and its interaction with diel cyclic temperature on liver phospholipids.

**Conclusion:**

Temperatures of 9 and 12.5 °C could elevate membrane fluidity and thickness in the liver and intestine of rainbow trout in freshwater, whereas no significant effects were found with diel temperature variations. After seawater acclimation, constant and diel cyclic temperatures significantly influenced the membrane fluidity and thickness of the liver and intestine. Compared with constant temperature, diel temperature variation (13 ± 2 °C) can enhance the adaptability of rainbow trout during seawater acclimation.

**Supplementary Information:**

The online version contains supplementary material available at 10.1186/s40850-021-00086-6.

## Background

Salmonids are one of the most economically farmed species worldwide. Million tons of Atlantic salmon (*Salmo salar*) in 2018 exceeded 2.4 million tons, while that of rainbow trout (*Oncorhynchus mykiss*) reached 0.8 million tons [1]. The surge in demand for salmonids in China is mainly driven by consumers’ preference for the taste and abundance of essential amino acids and polyunsaturated fatty acids (PLFAs) [2]. The discovery of the Yellow Sea Cold Water Mass opened up the possibility of offshore salmon mariculture [3, 4], one of the pivotal stages of this project is the ‘mountain-sea relay’ mode, in which salmon are hatched and bred until the juvenile stage in freshwater in mountainous areas, transferred to seawater, and fattened to commercial size. Previous research has reported that the seawater tolerance of salmonids can be influenced by life stage [5], photoperiod [6], salinity stimulation [7], nutritional status [8, 9], and temperature change [10, 11].

During adaptation to environmental changes (e.g., temperature and salinity), organisms alter the physical properties of the membrane, including fluidity, thickness, permeability, and viscosity, by restructuring the PLFA composition [12, 13]. These changes significantly influence the biological functions of the membrane [14]. Biological membranes are susceptible to changes in temperature and salinity. When the temperature decreases, fish increase the degree of unsaturation to maintain sufficient fluidity, and vice versa [15, 16]. When salinity increases, some fish increase membrane fluidity by increasing the proportion of unsaturated fatty acids [17].

The rainbow trout is one of the most important cultured aquatic species, both in China and worldwide, and can serve as an excellent model for the study of environmental physiology in fish [18–20]. Environmental changes induce adaptive alterations in fish tissues, and the restructuring of PLFA composition is an important step in regulating membrane function [21]. For instance, a decrease in temperature can increase membrane fluidity by increasing the degree of unsaturation in fish tissues, which has been demonstrated in alewives (*Alosa pseudoharengus*) [22], yellow perch (*Perca flavescens*) [23], steelhead trout (*O. mykiss*), and Atlantic salmon [15]. Similarly, fish living in seawater will also have a higher proportion of unsaturated fatty acids in tissue phospholipids, which can be used to maintain membrane function [24, 25]. To date, however, there has been comparatively little research focusing on the membrane response of salmonids during seawater transition. Nonetheless, determining whether salinity and temperature interactions affect the changes in PLFA composition in rainbow trout is of particular importance, as this can provide useful information regarding seawater acclimation and feeding strategies in salmonid aquaculture production.

Our research group has previously found that non-stressful low temperatures can enhance the osmoregulatory capacity of rainbow trout [26]. In the present study, we selected 16 °C as the baseline temperature based on the findings of Austreng et al. [27], who have reported the highest growth rates of rainbow trout at this temperature. Thereafter, two lower temperatures (9 and 12.5 °C) within the optimal temperature range were selected, and fish were subsequently exposed to diel cyclic temperatures of 13 ± 1 and 13 ± 2 °C, which were designed to mimic natural temperature variations within the Yellow Sea [28]. The aforementioned studies have reported that in terms of osmoregulation and branchial phospholipids, temperatures of 12.5 °C and 13 ± 2 °C are ideal for seawater acclimation in rainbow trout [11, 26]. However, the effects of seawater acclimation at different temperatures on the liver and intestinal phospholipids in rainbow trout are yet to be sufficiently determined. In this study, we accordingly aimed to acclimate rainbow trout at different temperatures and analyze changes in PLFA composition and seawater tolerance.

## Results

### Effects of constant temperature on liver phospholipid fatty acid composition (PLFA) during seawater acclimation

**Fig. 1:**
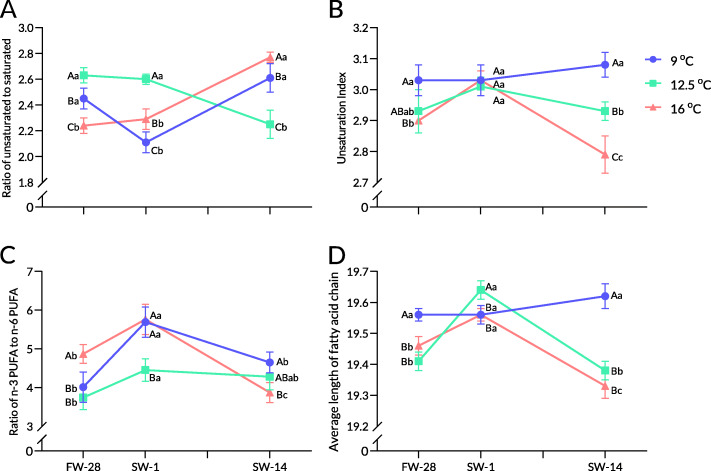
Liver PLFA compositions at different constant temperatures during seawater acclimation of rainbow trout. Note: The ratio of unsaturation (**A**), unsaturation index (**B**), ratio of n-3 PUFA to n-6 PUFA (**C**), and average length of fatty acid chain (**D**). Values represent the means of three replicates. Capital letters indicate intergroup differences at the same sampling point, whereas lowercase letters indicate differences at different sampling points in the same treatment

In trial 1, after rearing in freshwater for 4 weeks (FW-28), the liver PLFA composition of rainbow trout was significantly altered by different constant temperatures (Table 1). In treatments of 9 and 12.5 °C, the proportions of 16:0 and 18:0 significantly lowered the ratio of saturated fatty acid (SFA) in liver phospholipids. The proportion of monounsaturated fatty acids (MUFAs) in liver phospholipids was significantly lower at 16 °C than at 12.5 °C, but significantly higher than that at 9 °C, mainly caused by 18:1n9. The proportion of polyunsaturated fatty acids (PUFAs) in the liver phospholipids of rainbow trout was negatively correlated with temperature; the proportion of PUFAs at 9 °C was the highest, followed by those at 12.5 and 16 °C. The ratio of unsaturated to saturated fatty acid (U/S) at 9 and 12.5 °C was significantly higher than that at 16 °C, while the unsaturation index (UI) at 9 °C was significantly higher than that at 16 °C. The ratio of n-3 PUFA to n-6 PUFA (n3/n6) at 9 and 12.5 °C was significantly higher than that at 16 °C (Fig. 1A–D). In addition, the average chain length (ACL) of fatty acids at 9 °C was significantly higher than that in the other groups.

**Table 1:** Liver PLFA composition of rainbow trout in different constant temperatures during seawater acclimation

SP	FW-28			SW-1			SW-14		
Temperature	9 °C	12.5 °C	16 °C	9 °C	12.5 °C	16 °C	9 °C	12.5 °C	16 °C
Saturated fatty acid									
C14:0	1.11^Ab^	1.11^A^	0.82^B^	1.38^Aa^	0.83^B^	0.97^B^	1.04^b^	1.25	1.03
C16:0	17.09^Ba^	17.39^B^	18.95^Aa^	17.40^Aa^	17.55^A^	14.80^Bb^	15.53^Bb^	18.78^A^	14.56^Bb^
C17:0	0.44	0.42	0.41	0.37	0.35	0.60	0.57	0.52	0.49
C18:0	9.45^Ab^	7.40^Bb^	9.30^A^	11.29^Aa^	10.24^Ba^	9.21^C^	9.18^Ab^	9.25^Aa^	8.65^B^
C20:0	0.28	0.25	0.35^a^	0.20	0.19	0.24^ab^	0.22	0.29	0.18^b^
C22:0	0.05^b^	0.08	0.09^b^	0.18^Aa^	0.05^B^	0.06^Bb^	0.05^Bb^	0.08^B^	0.23^Aa^
C23:0	0.25^b^	0.52^a^	0.39^b^	0.63^a^	0.54^a^	0.70^a^	0.05^Bc^	0.06^Bb^	0.23^Ab^
C24:0	0.33^Ac^	0.41^AB^	0.60^Ab^	0.65^Bb^	0.63^B^	1.16^Aa^	1.08^Aa^	0.51^B^	1.15^Aa^
ƩSFA	29.00^Bb^	27.57^Cb^	30.9^Aa^	32.11^Aa^	30.38^Ba^	27.75^Cb^	27.72^Bc^	30.73^Aa^	26.51^Bb^
Monounsaturated fatty acid									
C14:1n5	0.73	0.60^b^	0.55^b^	0.54	0.62^b^	1.01^a^	1.09	1.33^a^	1.05^a^
C16:1n7	1.07^C^	2.18^Aa^	1.96^Bb^	1.21	1.71^b^	1.35^c^	1.24^B^	1.86^Bb^	3.29^Aa^
C17:1n7	0.25	0.20	0.14	0.13^b^	0.15	0.20	0.26^a^	0.17	0.21
C18:1n9	7.23^C^	11.55^Aa^	9.18^Bb^	7.33^B^	8.51^ABb^	9.79^Ab^	7.68^B^	7.48^Bc^	13.95^Aa^
C20:1n9	0.28	0.21	0.30	0.24	0.22	0.25	0.26	0.30	0.25
C22:1n9	0.44^a^	0.31	0.49	0.25^b^	0.23	0.33	0.05^Bc^	0.34^A^	0.41^A^
C24:1n9	3.05^Aa^	2.55^B^	3.16^A^	2.57^Bb^	2.44^B^	3.24^A^	3.39^Aab^	2.67^AB^	2.41^B^
ƩMUFA	13.04^C^	17.59^Aa^	15.78^Bb^	12.26^B^	13.87^Bb^	16.18^Ab^	13.98^B^	14.16^Bb^	21.57^Aa^
Polyunsaturated fatty acid									
C18:2n6	4.57^Ba^	5.25^Aa^	4.17^Cb^	3.61^b^	3.43^c^	4.24^ab^	4.58^ab^	4.56^b^	5.52^a^
C18:3n3	2.01^a^	2.02^a^	2.07	1.71^Bb^	1.64^Bc^	1.94^A^	2.11^Aa^	1.87^Bb^	2.03^A^
C18:3n6	0.23	0.19	0.21	0.17	0.11	0.14	0.18	0.15	0.14
C20:2n6	1.85^Aa^	1.09^AB^	0.82^B^	0.95^ABc^	0.87^B^	1.44^A^	1.52^b^	1.35	1.51
C20:3n3	2.07^Ab^	1.26^Bb^	1.19^B^	1.23^c^	2.04^a^	1.52	2.19^Aa^	1.57^Bb^	1.24^C^
C20:3n6	1.06^a^	1.60^a^	1.83^a^	0.66^b^	0.93^b^	0.83^b^	0.62^Bb^	1.05^Ab^	0.64^Bb^
C20:4n6	5.30^Aa^	4.26^Ba^	2.72^Cb^	3.73^Bc^	3.59^Bb^	4.76^Aa^	4.61^b^	4.42^a^	4.02^a^
C20:5n3	4.36^b^	4.14	4.39	5.40^Aa^	4.61^B^	4.63^B^	5.87^Aa^	4.71^B^	4.98^B^
C22:2n6	0.20^b^	0.17^b^	0.11^b^	0.08^Bc^	0.45^Aa^	0.34^Aa^	0.30^Aa^	0.09^Cb^	0.21^Bab^
C22:6n3	36.31^Ab^	34.85^Cb^	35.81^Ba^	38.09^Aa^	38.07^Aa^	36.23^Ba^	36.31^Ab^	35.34^Ab^	31.67^Bb^
ƩPUFA	57.96^Aa^	54.84^B^	53.32^Cb^	55.63^b^	55.75	56.07^a^	58.30^Aa^	55.11^B^	51.92^Cb^

Note: Values are means of 3 replications. Different lowercase letters indicate significant differences (*P* < 0.05) among different treatments at the same time, and different capital letters indicate significant differences at different times at the same treatment. Data containing mean ± SD are included in Additional file 2. *FW-28* End of growth trial, *SW-1* One day after salinity reached 30, *SW-4* Four days after salinity reached 30, *SW-7* Seven days after salinity reached 30, *SW-14* Fourteen days after salinity reached 30

On the first day after seawater acclimation (SW-1, the first day after salinity reached 30 ppt), the liver PLFA composition in rainbow trout was significantly altered (Table 1). The proportion of SFA in liver phospholipids significantly decreased at 16 °C, whereas that at 9 and 12.5 °C increased, which was significantly higher than that at 16 °C. The total MUFA at 12.5 °C significantly decreased, while those in other groups remained unchanged. The proportion of PUFAs at 9 °C increased to 55.75%, close to the level at 12.5 °C. Figure 1 shows a significantly low U/S at 9 °C and a significantly high ACL at 12.5 °C, but no significant difference in UI among groups.

Two weeks after seawater acclimation, the total SFA at 9 °C significantly decreased and was significantly lower than that at 12.5 °C (Table 1). The total MUFA at 16 °C increased considerably and was significantly higher than that in the other groups. Consequently, the UI at 9 and 16 °C increased and was significantly higher than that at 12.5 °C. These changes resulted in significantly higher UI, U/S, and ACL at 9 °C (Fig. 1A-D).

### Effects of constant temperature on intestinal PLFA composition during seawater acclimation

**Fig. 2:**
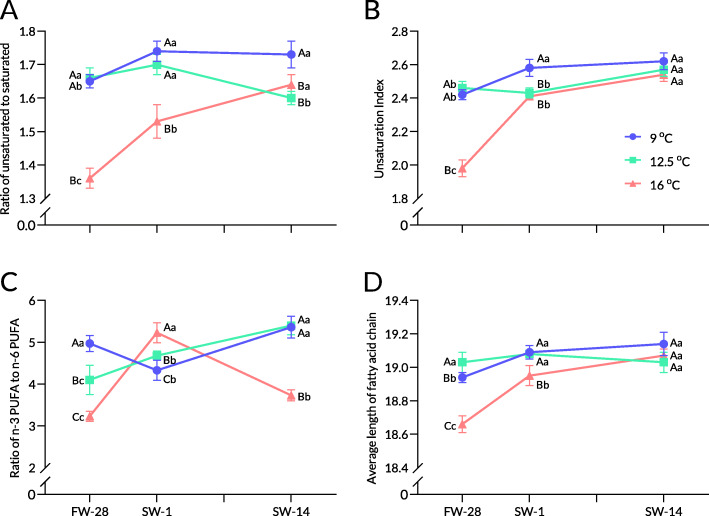
Intestinal PLFA compositions at different constant temperatures during seawater acclimation of rainbow trout. Note: The ratio of unsaturation (**A**), unsaturation index (**B**), ratio of n-3 PUFA to n-6 PUFA (**C**), and average length of fatty acid chain (**D**). Values represent the means of three replicates. Capital letters indicate intergroup differences at the same sampling point, whereas lowercase letters indicate differences at different sampling points in the same treatment

In trial 1, after rearing in freshwater for 4 weeks, the intestinal PLFA composition in rainbow trout was significantly altered (Table 2). The total SFA at 9 and 12.5 °C decreased significantly, while the total PUFAs at 9 and 12.5 °C were significantly higher at 16 °C. The total MUFA at 12.5 °C significantly decreased and was significantly lower than that at 16 °C. These changes resulted in significantly higher U/S, UI, and ACL at 9 and 12.5 °C than at 16 °C (Fig. 2A-D). The n3/n6 ratio was positively associated with temperature.

**Table 2:** Intestinal PLFA composition of rainbow trout in different constant temperatures during seawater acclimation

SP	FW-28			SW-1			SW-14		
Temperature	9 °C	12.5 °C	16 °C	9 °C	12.5 °C	16 °C	9 °C	12.5 °C	16 °C
Saturated fatty acid									
C14:0	2.73^B^	2.26^C^	3.32^Aa^	2.48	1.67	2.20^ab^	2.50	2.49	1.63^b^
C16:0	22.31	22.06	23.99^a^	22.37	21.55	23.58^a^	21.16^B^	23.10^A^	21.38^Bb^
C17:0	0.78^B^	0.49^Bb^	1.25^Aa^	0.81^A^	0.75^ABa^	0.58^Bb^	0.62	0.54^b^	0.67^b^
C18:0	10.27^AB^	11.51^A^	10.22^Bb^	9.29^B^	11.69^A^	11.44^Aab^	10.45	11.23	12.57^a^
C20:0	0.29	0.32	0.45^a^	0.25	0.22	0.31^ab^	0.17	0.26	0.17^b^
C22:0	0.18^A^	0.10^Ba^	0.11^B^	0.14	0.16^a^	0.18	0.11	0.05^b^	0.16
C23:0	0.12^AB^	0.07^B^	0.13^A^	0.13^A^	0.04^B^	0.11^A^	0.12	0.07	0.11
C24:0	1.04^B^	0.76^C^	2.91^Aa^	1.05	0.96	1.19^b^	1.54	0.68	1.16^b^
ƩSFA	37.71^B^	37.59^B^	42.38^Aa^	36.53^B^	37.04^B^	39.60^Ab^	36.66	38.42	37.84^c^
Monounsaturated fatty acid									
C14:1n5	1.64^Ba^	1.06^C^	2.93^Aa^	0.86^Cb^	1.19^B^	1.61^Ab^	0.93^b^	1.05	0.62^c^
C16:1n7	1.86	1.51	1.51	2.40^A^	1.26^B^	1.39^B^	2.13^A^	1.72^AB^	1.38^B^
C17:1n7	0.53^a^	0.47	0.45	0.29^Bb^	0.45^A^	0.19^B^	0.34^b^	0.40	0.34
C18:1n9	10.85^a^	9.39^a^	10.21^a^	8.20^Bb^	10.68^Aa^	9.47^ABab^	8.32^b^	7.42^b^	8.86^b^
C20:1n9	0.23^b^	0.22	0.35^a^	0.21^b^	0.17	0.19^b^	0.35^a^	0.23	0.27^ab^
C22:1n9	0.60	0.54^b^	0.85^a^	0.71	0.77^a^	0.64^b^	0.52	0.57^b^	0.52^b^
C24:1n9	2.49	3.22^a^	3.24^a^	3.01	3.49^a^	2.53^ab^	2.39^A^	2.55^Ab^	1.63^Bb^
ƩMUFA	18.19^ABa^	16.42^Ba^	19.54^Aa^	15.67^Bb^	18.02^Aa^	16.01^Bb^	14.98^b^	13.93^b^	13.62^c^
Polyunsaturated fatty acid									
C18:2n6	3.83^Bb^	5.52^Aa^	4.96^Ab^	5.07^a^	4.50^ab^	4.19^b^	3.93^b^	3.75^b^	7.01^a^
C18:3n3	2.75^AB^	2.30^B^	3.80^A^	2.63	2.85	3.12	2.82	3.30	2.50
C18.3n6	0.34^a^	0.22	0.43	0.15^b^	0.16	0.18	0.12^b^	0.15	0.24
C20:2n6	0.79	0.70^a^	0.78	0.67	0.72^a^	0.72	0.79	0.56^b^	0.74
C20:3n3	1.01	1.11	0.97	0.75^B^	1.36^A^	0.82^B^	1.17	1.05	0.70
C20:3n6	0.55	0.67	0.73^a^	0.56^A^	0.47^A^	0.31^Bb^	0.45	0.64	0.53^a^
C20:4n6	2.49	2.45	2.61	3.04	2.60	2.30	2.94	2.77	2.29
C20:5n3	1.76^Bb^	2.94^Aa^	2.17^AB^	2.90^ab^	2.39^b^	2.81	3.70^a^	3.70^a^	2.74
C22:2n6	0.33^a^	0.18^b^	0.38^a^	0.15^Bb^	0.31^Aa^	0.25^Aab^	0.16^b^	0.23^ab^	0.19^b^
C22:6n3	30.24^Ab^	29.90^A^	21.25^Bb^	31.89^ab^	29.58	29.71^a^	32.28^a^	31.50	31.61^a^
ƩPUFA	44.09^Ab^	45.99^Aab^	38.07^Bc^	47.80^Aa^	44.94^Bb^	44.39^Bb^	48.35^a^	47.65^a^	48.54^a^

Note: Values are means of 3 replications. Different lowercase letters indicate significant differences (*P* < 0.05) among different treatments at the same time, and different capital letters indicate significant differences at different times at the same treatment. Data containing mean ± SD are included in Additional file 2. *FW-28* End of growth trial, *SW-1* One day after salinity reached 30, *SW-4*, Four days after salinity reached 30, *SW-7* Seven days after salinity reached 30, *SW-14* Fourteen days after salinity reached 30

The total SFA at 16 °C significantly decreased on the first day after seawater acclimation. The total MUFA at 9 and 16 °C significantly decreased, whereas that at 12.5 °C increased and was significantly higher than in other groups. The total PUFA at 9 and 16 °C significantly increased, while that at 12.5 °C was relatively steady. Consequently, significant increases in n3/n6, U/S, UI, and ACL were recorded at 16 °C.

Two weeks after seawater acclimation (SW-14, the 14th day after salinity reached 30 ppt), the total SFA at 16 °C decreased, while the other groups showed no significant difference (Table 2). The total MUFA in all groups decreased significantly. Accordingly, the total PUFA in all groups increased but showed no difference. Thus, the UI at 9 °C was significantly higher than that in other groups, whereas no difference was found in UI and ACL in liver phospholipids in rainbow trout (Fig. 2A-D).

### Effects of diel cyclic temperature on liver PLFA composition during seawater acclimation

**Fig. 3:**
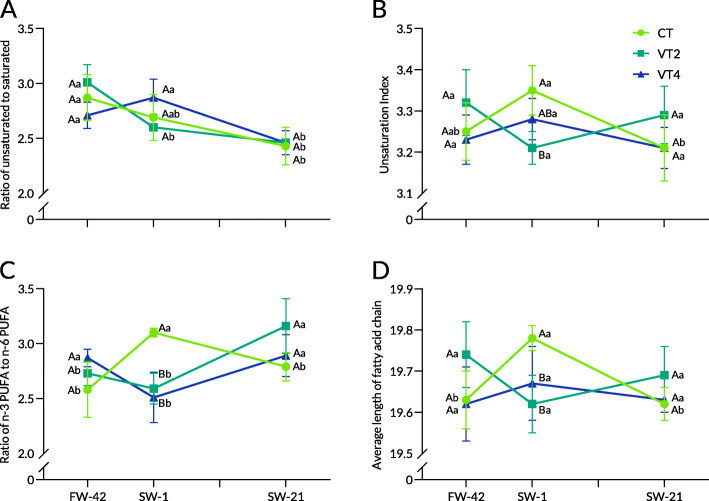
Liver PLFA compositions at diel cyclic temperatures during seawater acclimation in rainbow trout. Note: The ratio of unsaturation (**A**), unsaturation index (**B**), ratio of n-3 PUFA to n-6 PUFA (**C**), and average length of fatty acid chain (**D**). Values represent the mean of four replicates. Capital letters indicate intergroup differences at the same sampling point, whereas lowercase letters indicate differences at different sampling points in the same treatment

In trial 2, on the final day of the growth trial (FW-42), the proportion of 16:0 in the treatment of 13 °C (CT) was significantly decreased, while that of 20:4n6 in the treatment of 13 ± 1 °C (VT2) and 13 ± 2 °C (VT4) remained steady (Table 3). However, no significant change was found in total SFA, MUFA, and PUFA among the groups. Therefore, there was no difference among groups in terms of U/S, UI, and n3/n6, except for a significantly higher ACL in VT2 (Fig. 3A-D).

**Table 3:** Liver PLFA composition (%) in rainbow trout at different sampling points at diel cyclic temperatures

SP	FW-42			SW-1			SW-21		
Temp	CT	VT2	VT4	CT	VT2	VT4	CT	VT2	VT4
Saturated fatty acid									
C14:0	0.96^b^	1.00^b^	0.97^b^	1.18^a^	1.16^a^	1.13^a^	1.07^ab^	1.19^a^	1.11^a^
C16:0	17.26^AB^	15.98^Bc^	17.90^Aa^	16.95	17.40^b^	16.50^b^	18.41	18.77^a^	18.26^a^
C17:0	0.61^ab^	0.53	0.71^a^	0.44^ABb^	0.57^A^	0.26^Bb^	0.74^a^	0.61	0.67^a^
C18:0	6.23^b^	6.21^b^	6.27^c^	7.66^a^	7.63^a^	7.35^b^	8.09^a^	7.70^a^	8.20^a^
C20:0	0.21	0.19^a^	0.19	0.13	0.13^ab^	0.14	0.15	0.09^b^	0.15
C22:0	0.12	0.09	0.16	0.08	0.12	0.08	0.10	0.09	0.08
C24:0	0.52^Bb^	0.94^A^	0.79^ABa^	0.76^a^	0.82	0.38^b^	0.63^Aab^	0.42^B^	0.48^ABb^
ƩSFA	25.92^b^	24.94^b^	27.00^b^	27.20^ab^	27.82^a^	25.84^b^	29.19^a^	28.87^a^	28.96^a^
Monounsaturated fatty acid									
C14:1n5	0.79^b^	0.78	0.98^b^	0.60^b^	0.92	0.59^c^	1.42^a^	1.01	1.28^a^
C16:1n7	2.61^a^	2.54^a^	2.70^a^	1.42^b^	2.04^ab^	2.14^ab^	1.23^b^	1.34^b^	1.59^b^
C17:1n7	0.23	0.19	0.20	0.23^A^	0.20^A^	0.17^B^	0.19	0.22	0.21
C18:1n9	2.01^a^	2.09^a^	2.11^a^	1.51^Bb^	1.68^ABb^	1.86^Aa^	1.60^b^	1.55^b^	1.56^b^
C20:1n9	0.20	0.17	0.15	0.18	0.16	0.20	0.18	0.13	0.17
C22:1n9	0.08	0.05	0.08	0.06	0.05	0.05	0.06	0.06	0.04
C24:1n9	2.81	2.91	2.62^b^	2.88	2.75	2.71^b^	3.18	3.01	3.19^a^
ƩMUFA	8.72^a^	8.72^a^	8.84^a^	6.88^Bb^	7.80^Ab^	7.72^Ab^	7.88^a^	7.32^b^	8.04^b^
Polyunsaturated fatty acid									
C18:2n6	12.89^a^	12.86^a^	12.92^a^	10.22^Bb^	12.00^Aa^	13.23^Aa^	9.18^b^	8.89^b^	9.58^b^
C18:3n3	0.10	0.10	0.10^ab^	0.15^A^	0.10^B^	0.08^Bb^	0.14	0.14	0.13^a^
C18:3n6	0.16	0.16	0.13	0.13	0.14	0.15	0.15	0.13	0.10
C20:2n6	1.26^Aba^	1.48^Aa^	1.12^B^	1.18^a^	1.21^ab^	1.36	0.79^b^	0.97^b^	1.18
C20:3n3	0.91^b^	0.84^b^	0.84^b^	1.33^Aa^	0.96^Bb^	0.94^Bb^	1.24^a^	1.28^a^	1.21^a^
C20:3n6	1.93^A^	1.50^Bab^	1.29^Bb^	2.03	1.83^a^	2.06^a^	1.82^A^	1.14^Bb^	1.54^ABb^
C20:4n6	2.92^Ab^	2.78^Ac^	1.85^Bc^	3.37^b^	3.53^b^	3.06^b^	5.19^a^	4.93^a^	4.63^a^
C20:5n3	2.69^b^	2.52	2.12^b^	3.01^ab^	2.90	2.80^a^	3.64^Aa^	2.60^B^	2.92^Ba^
C22:2n6	0.34	0.33	0.38	0.23	0.37	0.28	0.23	0.29	0.30
C22:6n3	42.16^b^	43.78^a^	43.4^a^	44.29^Aa^	41.33^Cb^	42.47^Bab^	40.54^Bb^	43.45^Aa^	41.39^Bb^
ƩPUFA	65.36	66.34^a^	64.16^b^	65.92	64.38^ab^	66.44^a^	62.93	63.81^b^	63.00^b^

Note: Values represent the mean of 4 replicates. Different lowercase letters indicate significant differences (*P* < 0.05) among different treatments at the same time, and different capital letters indicate significant differences at different times at the same treatment. Data containing mean ± SD are included in Additional file 2. *FW-42* End of growth trial, *SW-1* One day after salinity reached 30, *SW-21* 21 days after salinity reached 30, *SFA* Saturated fatty acid, *MUFA* Monounsaturated fatty acid, *PUFA* Polyunsaturated fatty acid

At SW-1, the total SFA of liver phospholipids in CT and VT2 increased significantly, while VT4 showed no significant change (Table 3). An overall decrease in total MUFA was detected in all groups, and the CT group showed significantly lower total MUFA than the VT2 and VT4 groups. Furthermore, the proportion of 22:6n3 was significantly lower in VT2 and VT4 than in CT, while total PUFA showed no difference among the groups. These changes resulted in a significantly higher UI in the CT than in the VT2. ACL and n3/n6 were significantly higher in CT than in VT2 and VT4 (Fig. 3A-D).

In trial 2, 3 weeks after seawater acclimation (SW-21, the 21st day after salinity reached 30 ppt), the total SFA in all groups increased significantly and showed no difference in liver phospholipids of rainbow trout (Table 3). The total MUFA in CT increased to 7.88%, while in VT2 and VT4 it was comparatively steady. The total PUFA in VT2 and VT4 decreased significantly and showed no differences among the groups. The PLFA-related parameters showed no significant differences between the groups.

### Effects of diel cyclic temperature on intestinal PLFA composition during seawater acclimation

**Fig. 4:**
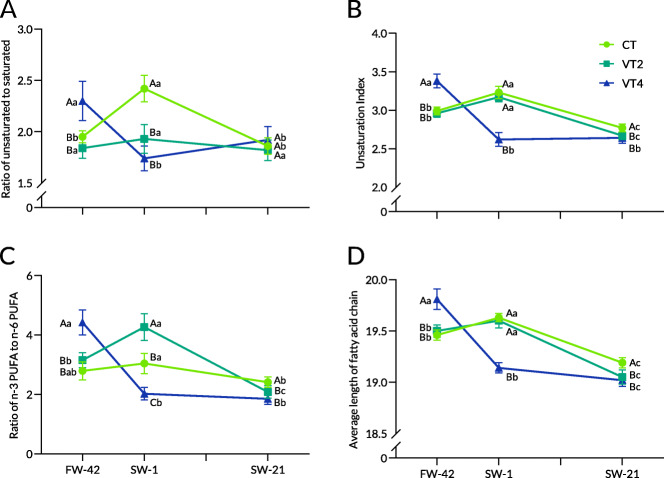
Intestinal PLFA compositions at diel cyclic temperatures during seawater acclimation in rainbow trout. Note: The ratio of unsaturation (**A**), unsaturation index (**B**), ratio of n-3 PUFA to n-6 PUFA (**C**), and average length of fatty acid chain (**D**). Values represent the mean of four replicates. Capital letters indicate intergroup differences at the same sampling point, whereas lowercase letters indicate differences at different sampling points in the same treatment

In trial 2, after 6 weeks in freshwater, the intestinal PLFA composition of rainbow trout was significantly influenced by diel cyclic temperature (Table 4). The total SFA in VT4 was significantly lower than that in CT and VT2. A significantly high proportion of 22:6n3 was recorded in VT4, resulting in a significantly higher total PUFA in VT4. Correspondingly, the U/S, UI, n3/n6, and ACL in VT4 were significantly higher than those in CT and VT2 (Fig. 4A-D).

**Table 4:** Intestinal PLFA composition (%) in rainbow trout at different sampling points at diel cyclic temperatures

SP	FW-42			SW-1			SW-21		
Temp	CT	VT2	VT4	CT	VT2	VT4	CT	VT2	VT4
Saturated fatty acid									
C14:0	1.27^b^	1.14^b^	1.02^c^	1.28^b^	1.20^b^	1.25^b^	1.50^a^	1.72^a^	1.67^a^
C16:0	19.84^ABa^	21.32^A^	18.76^B^	16.96^Bb^	20.7^A^	20.46^A^	20.95^a^	21.37	20.20
C17:0	0.62	0.73	0.63	0.82	0.68	1.01	0.88	1.11	0.85
C18:0	11.07^Aa^	10.36^A^	8.69^Bc^	9.19^Bb^	10.41^B^	12.59^Aa^	10.67^a^	9.81	10.49^b^
C20:0	0.27	0.38^a^	0.33^a^	0.26^AB^	0.29^Aab^	0.21^Bb^	0.22	0.23^b^	0.22^b^
C22:0	0.11	0.14	0.13	0.15	0.10	0.14	0.11	0.19	0.12
C24:0	0.77^B^	1.20^Aa^	0.81^B^	0.60^B^	0.81^Ab^	0.86^A^	0.74	0.90^ab^	0.77
ƩSFA	33.94^Aa^	35.28^A^	30.37^Bb^	29.26^Bb^	34.19^A^	36.51^Aa^	35.08^a^	35.32	34.31^a^
Monounsaturated fatty acid									
C14:1n5	0.55^c^	0.74^c^	0.69^b^	1.00^b^	1.00^b^	1.41^a^	1.37^a^	1.28^a^	1.28^a^
C16:1n7	1.12^b^	0.96^b^	0.96^b^	1.30^b^	0.90^b^	1.40^b^	2.22^a^	2.78^a^	2.97^a^
C17:1n7	0.34	0.30	0.26^b^	0.45^AB^	0.25^B^	0.59^Aa^	0.34	0.40	0.37^b^
C18:1n9	2.07	2.22^b^	1.87^b^	2.08^B^	1.89^Bc^	2.57^Aa^	2.25^B^	2.63^Aa^	2.70^Aa^
C20:1n9	0.28	0.33	0.28	0.26	0.26	0.33	0.27	0.33	0.31
C22:1n9	0.07	0.10	0.08	0.07	0.09	0.08	0.10	0.09	0.10
C24:1n9	2.64	2.54^a^	2.34^ab^	2.18	2.06^b^	2.69^a^	2.36^A^	1.95^Bb^	1.73^Bb^
ƩMUFA	7.07^b^	7.20^b^	6.46^b^	7.32^Bb^	6.46^Bb^	9.07^Aa^	8.90^a^	9.47^a^	9.46^a^
Polyunsaturated fatty acid									
C18:2n6	10.58^A^	9.43^Ab^	7.43^Bb^	11.7^B^	7.06^Cc^	13.56^Aa^	11.90^C^	13.35^Ba^	14.86^Aa^
C18:3n3	0.12	0.11	0.11^b^	0.1	0.09	0.11^b^	0.09	0.15	0.20^a^
C18:3n6	0.16^b^	0.16^b^	0.15^b^	0.26^ab^	0.13^b^	0.19^b^	0.36^a^	0.58^a^	0.67^a^
C20:2n6	0.80^A^	0.51^Bb^	0.59^ABb^	0.61	0.55^b^	0.76^a^	0.71	0.81^a^	0.82^a^
C20:3n3	0.79	0.74^b^	0.69	0.73	0.69^b^	0.88	0.93	0.98^a^	0.96
C20:3n6	1.32^Aa^	0.89^B^	0.91^B^	0.89^b^	0.84	1.00	0.91^b^	0.90	0.96
C20:4n6	3.28^a^	3.20	3.02	2.68^b^	3.11	2.86	2.99^ab^	2.72	2.76
C20:5n3	2.36^b^	2.06^b^	2.13^b^	2.34^b^	2.14^b^	2.45^b^	3.03^a^	3.19^a^	3.24^a^
C22:2n6	0.37	0.42	0.43	0.40	0.46	0.42	0.38	0.51	0.49
C22:6n3	39.21^Bb^	40.01^Bb^	47.71^Aa^	43.71^Aa^	44.27^Aa^	32.16^Bb^	34.71^Ac^	32.02^Bc^	31.28^Bb^
ƩPUFA	58.99^Bb^	57.52^Bb^	63.17^Aa^	63.42^Aa^	59.34^Ba^	54.42^Bb^	56.02^c^	55.21^c^	56.23^b^

Note: Values represent the mean of 4 replicates. Different lowercase letters indicate significant differences (*P* < 0.05) among different treatments at the same time, and different capital letters indicate significant differences at different times at the same treatment. Data containing mean ± SD are included in Additional file 2. *FW-42* End of growth trial, *SW-1* One day after salinity reached 30, *SW-21* 21 days after salinity reached 30, *SFA* Saturated fatty acid, *MUFA* Monounsaturated fatty acid, *PUFA* Polyunsaturated fatty acid

At SW-1, significant decreases in the proportions of 16:0 and 18:0 were detected in CT in the intestinal phospholipids of rainbow trout (Table 4). The total MUFA in VT4 significantly increased and was significantly higher than that in CT and VT2. The proportion of 22:6n3 in CT and VT2 increased significantly, whereas that in VT4 decreased. Consequently, the total PUFA in CT was significantly higher than that in VT2 and VT4 (Fig. 4A-D). These changes caused significantly higher U/S in CT than in VT2 and VT4, whereas the UI and ACL in VT4 were significantly lower than those in the other groups.

At SW-21, the intestinal phospholipids contained significantly lower proportions of 18:1n9 and 18:2n6 in CT than in VT2 and VT4, whereas the proportion of 22:6n3 in CT was significantly higher than that in VT2 and VT4 (Table 4). However, no significant differences were observed in total SFA, MUFA, and PUFA among the groups. The UI, n3/n6, and ACL in CT were significantly higher than those in VT2 and VT4 (Fig. 4A-D).

### A two-way analysis of variance

In trial 1, the two-way analysis of variance (ANOVA) indicated that both the liver and intestine in rainbow trout were significantly influenced by constant temperature, seawater acclimation, and their interaction (see Additional file 3). In trial 2, the diel cyclic temperature had no significant effect on liver PLFA composition, whereas seawater acclimation significantly influenced the total SFA, n3/n6, U/S, UI, and ACL in the liver PLFA composition. Therefore, the total SFA, n3/n6, U/S, UI, and ACL were significantly influenced by the interaction between the diel cyclic temperature and seawater acclimation. In the intestinal phospholipids, all selected parameters were significantly affected by diel cyclic temperature, seawater acclimation, and their combination, except total MUFA, which was free of the effect of temperature.

### Principal component analysis

In the present study, principal component analysis (PCA) was performed to reveal tissue differences in rainbow trout at different temperature regimes and seawater acclimation stages. Based on the results of the two-way ANOVA, 16 variables (14:0, 16:0, 18:0, 16:1, 18:1n9, 24:1, 18:2n6, 20:2n6, 20:3n3, 20:4n6, 20:5n3, and 22:6n3, U/S, UI, n3/n6, and ACL) were selected for the liver of rainbow trout, while nine variables (14:0, 18:0, 14:1, 16:1, 18:1n9, 18:2n6, 18:3n3, 20:5n3, 22:6n3, U/S, UI, n3/n6, and ACL) were selected for the intestine.

**Fig. 5:**
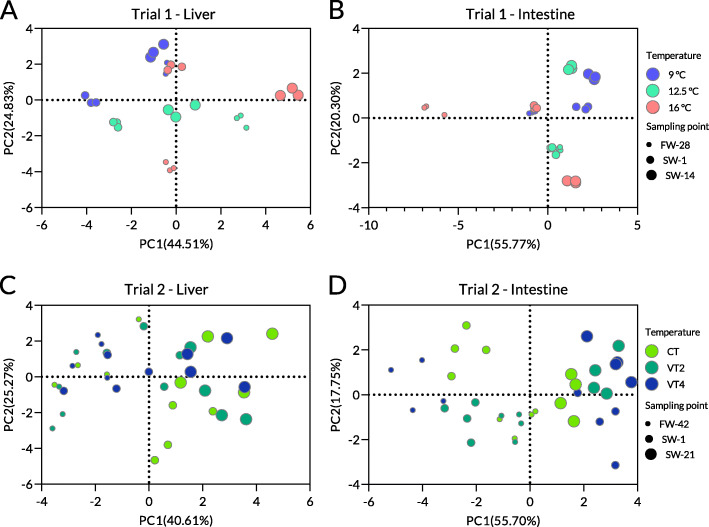
Score plots of liver and intestinal PLFA composition using PCA analysis. Note: Liver (**A**) and intestine (**B**) of rainbow trout at different constant temperatures, and liver (**C**) and intestine (**D**) of rainbow trout at diel cyclic temperature regimes, using principal component analysis

In trial 1, the first two principal components (PCs) with eigenvalues of 6.68 and 3.72 were found to explain 69.34% of the overall variability. The eigenvectors of 16:1, 18:0, 18:1n9, 18:2n6, 22:6n3, U/S, UI, n3/n6, and ACL were greater than 0.4, indicating a strong effect on the PCs. The score plots distinguished the liver PLFA composition of rainbow trout at different constant temperatures and seawater acclimation stages (Fig. 5A). PCA analysis of the intestinal PLFA composition yielded two PCs with eigenvalues of 6.70 and 2.44, explaining 76.06% of the dataset variance. The eigenvectors for 14:0, 14:1, 18:1n9, 18:3n3, 20:5n3, 22:6n3, U/S, UI, n3/n6, and ACL were greater than 0.4, resulting in a difference in the intestinal PLFA composition of rainbow trout at different temperatures and seawater acclimation stages (Fig. 5B).

In trial 2, two of three PCs were selected for PCA on liver PLFA composition, and their eigenvalues were 5.69 and 3.54 and explained 65.88% of the total variability. The eigenvectors for 16:1, 16:0, 18:2n6, 18:1n9, 18:0, 20:5n3, 20:3n3, n3/n6 and U/S exceeded 0.4. However, individuals of liver PLFAs clustered only at FW-42 and SW-21, instead of temperature regimes (Fig. 5C). Analysis of the intestinal PLFA composition produced two PCs with eigenvalues of 6.68 and 2.13, explaining 73.45% of the accumulated variability. Eigenvectors of more than 0.4 included 16:0, 18:2n6, 18:1n9, 18:0, 20:5n3, 22:6n3, 24:1, n3/n6, U/S, UI, and ACL, contributing to the comparatively high differentiation in intestinal phospholipids of rainbow trout influenced by diel cyclic temperature and seawater acclimation (Fig. 5D).

## Discussion

### Effects of constant temperature on the liver and intestinal PLFA composition in rainbow trout

To adapt to different temperatures, fish can restructure the membrane phospholipids of tissues to maintain proper metabolism and physiological homeostasis [29–31]. In the constant temperature experiment, the two-way ANOVA indicated a significant effect of temperature on the liver and intestinal phospholipids in rainbow trout. The higher U/S and UI of the liver and intestinal phospholipids at 9 and 12.5 °C implied an increase in membrane fluidity. Similarly, when the temperature decreased from 29 to 22 °C, the membrane fluidity of the liver in the European seabass (*Dicentrarchus labrax*) significantly increased [32]. Leray et al. [33] reported that rainbow trout (250 g) living at 12 °C showed a significantly higher degree of unsaturation in intestinal phospholipids than at 17 °C. Under low-temperature conditions, regulation of the degree of unsaturation of phospholipids is essential for maintaining the proper physical properties (fluidity, permeability, and viscosity) of cellular and organelle membranes [13, 34]. In contrast, increased membrane fluidity of the liver and intestine helps increase the transmembrane efficiency and activity of membrane-bound enzymes, enhancing the ability to absorb and utilize nutrients [35]. This adaptation alleviates the adverse effects of low temperatures, which limits the metabolic rate in fish [29, 31].

### Effects of constant temperature on the liver and intestinal PLFA composition in rainbow trout during seawater acclimation

Notably, on the first day after seawater acclimation in trial 1, the UI of liver phospholipids in rainbow trout at different temperatures changed significantly and converged to a similar level, while individuals from all groups clustered at SW-1 in the PCA. The results indicated that the effect of seawater acclimation was more significant than that of temperature and that the tissue membranes of rainbow trout adjust for acclimation to seawater. Similarly, Borlongan et al. [36] found that milkfish (*Chanos chanos* Forsskal) contained a significantly higher degree of unsaturation of liver phospholipid in seawater than in freshwater, indicating that fish can respond to salinity changes by altering membrane properties. In trial 1, 2 weeks after seawater acclimation, the UI and ACL at 12.5 and 16 °C changed significantly and reverted to levels before seawater acclimation, demonstrating that fish had basically completed the acclimation to salinity change. However, the liver PLFA composition of rainbow trout at 9 °C was significantly altered after seawater acclimation, but the fluidity remained unchanged, indicating that temperature had a greater influence than salinity change. Consequently, a temperature of 12.5 °C would appear to be more suitable with respect to liver membrane composition in rainbow trout, and thereby facilitates a rapid response during seawater acclimation.

In trial 1, on the first day after seawater acclimation, the U/S, UI, n3/n6, and ACL of intestinal phospholipids in the 16 °C group significantly increased and reached a level close to those in the 9 and 12.5 °C groups, indicating a significant alteration in fluidity, permeability, and thickness of the intestinal membrane. On the one hand, fish consume a large amount of energy for osmoregulation during seawater entry and can improve energy conversion efficiency by increasing membrane fluidity of the intestine to increase the efficiency of cross-membrane transportation and activities of membrane-bound enzymes [37, 38]. Brijs et al. [39] reported a dramatic increase in intestinal contractile activity in rainbow trout during seawater exposure, increasing the efficiency of absorptive processes. On the other hand, when fish are acclimated to seawater, ingested water and ions are absorbed by the intestine to maintain osmotic homeostasis [40]. Genz et al. [37] found a significant decrease in water, Na^+^, and Cl^−^ in intestinal fluids after rainbow trout acclimated to seawater, suggesting an enhanced absorption rate of the intestinal membrane. In the present study, the intestinal PLFA composition of rainbow trout showed no significant difference at SW-14, indicating that they had adapted to salinity changes. Overall, our findings indicate that temperatures between 9 and 12.5 °C are preferable for the seawater acclimation of rainbow trout, given that temperatures within this range tend to facilitate better adaptation.

### Effects of diel cyclic temperature on the liver and intestinal PLFA composition in rainbow trout

In trial 2, the liver phospholipids in rainbow trout showed no difference in U/S and UI. The two-way ANOVA and PCA analyses demonstrated that only seawater acclimation significantly influenced intestinal PLFA composition. This is because a) the liver of rainbow trout might have adapted to the diel cyclic changes in temperature after a 6-week stocking period under different temperature variations, and b) the liver of rainbow trout contains large amounts of long-chain PUFAs, suggesting better adaptability to temperature changes due to higher membrane fluidity.

The U/S, UI, and ACL in intestinal phospholipids of rainbow trout in VT4 were significantly higher than those at constant temperature, indicating an increase in membrane fluidity of the intestine. PCA results demonstrated that intestinal phospholipids in rainbow trout were more susceptible to temperature variations than liver phospholipids. Under diel cyclic temperature variation conditions, feed intake of fish will increase, and therefore the absorption efficiency of the intestinal membrane will be enhanced [41, 42]. Thomas et al. [43] reported that coho salmon (*Oncorhynchus kisutch*) under diel temperature cycles increased energy reserves by enhancing energy utilization efficiency. Accordingly, a diel temperature variation of 4 °C would appear to be beneficial with respect to intestinal absorption in rainbow trout.

### Effects of diel cyclic temperature on the liver and intestinal PLFA composition in rainbow trout during seawater acclimation

After the initial day of seawater acclimation in trial 2, we detected significant increases in the UI and ACL of liver phospholipids in rainbow trout, thereby implying a potential increase in the fluidity and thickness of the liver membrane, which are considered to be adaptations indicative of acclimation of the liver to seawater. Enhanced fluidity of the liver membrane is beneficial for the utilization of nutrients and can increase the synthesis and secretion of antioxidants [44, 45]. In trial 2, the liver PLFA composition of rainbow trout at VT4 was relatively stable, implying that diel cyclic temperatures could improve salinity tolerance to some extent. Similarly, after acclimation to periodic temperature variations for 3 days, the tolerance to acute heat stress in Atlantic salmon significantly improved [46]. Hokanson et al. [47] reported that rainbow trout subjected to daily temperature variations showed a higher growth rate and survival than those living at a constant temperature (15 °C). Our data showed that the liver PLFA compositions in all treatments were steady with no difference, suggesting that the adaptation to seawater had completed. Nonetheless, during seawater acclimation, the liver membrane of rainbow trout is more stable at diel cyclic temperatures.

In trial 2, on the first day after seawater acclimation, PCA analysis indicated that clusters appeared at different stages of seawater acclimation, demonstrating that intestinal phospholipids were more susceptible to salinity changes. Therefore, the U/S and UI of intestinal phospholipids significantly increased in CT but decreased in VT4, lowering the fluidity, permeability, and thickness of the intestinal membrane. These alterations represent adaptive changes in the intestine to counteract the salinity change in rainbow trout [48]. Decreased membrane fluidity can stimulate the activity of Na^+^-K^+^ ATPase located on the intestinal membrane and consequently contribute to osmotic homeostasis in fish [13, 49, 50]. In trial 2, the intestinal PLFA composition in all groups stabilized, implying that the fish had adapted to the salinity change. Moreover, fish under diel temperature variation up to 4 °C showed faster adaptation, proving that diel cyclic temperature can enhance the adaptability of rainbow trout to seawater acclimation.

## Conclusions

In this study, different constant temperatures significantly influenced the liver and intestinal PLFA composition in rainbow trout. The degree of unsaturation and average chain length of fatty acids in the liver and intestinal membrane significantly increased at 9 and 12.5 °C, respectively. Following the acclimation to seawater, the liver and intestinal PLFA compositions were initially influenced by salinity change, with those at 12.5 °C showing less fluctuations, although fish at all selected temperatures showed stable PLFA composition after 2 weeks. At different diel cyclic temperatures, the intestinal membrane of rainbow trout at 13 ± 2 °C had a significantly increased degree of unsaturation. During seawater acclimation, the liver’s PLFA composition was more stable, while the PLFA composition of the intestine responded to salinity changes more rapidly. Therefore, different constant temperatures significantly influenced the liver and intestinal phospholipids in rainbow trout, while the appropriate diel cyclic temperature can improve the adaptability to seawater acclimation in the liver and intestine of rainbow trout.

## Methods

### Animal husbandry

Diploid rainbow trout were purchased from Wanzefeng Fishery Company (Rizhao, China) and transported to the Key Laboratory of Mariculture at the Ocean University of China (Qingdao, China). Fish were reared in aquariums and acclimated to an experimental environment for 2 weeks, where the water temperature was maintained at optimal levels (16 ± 0.5 °C) by a semi-recirculating system equipped with refrigeration and aeration facilities. Throughout the experiment, rainbow trout were fed to satiation with commercial dry feed by hand (Qihao Biotechnology Company, Qingdao, China) twice daily (0800 and 1800 h). All fish stocking and experimental procedures were performed in accordance with the ethical approval of the Ocean University of China and National Experimental Management Regulations in China and were approved by the Animal Experimentation Committee of the Ocean University of China (Reference Number, 2018–12). All animal studies were performed in accordance with the ARRIVE guidelines [51]. The fatty acid composition of the feed is shown in Additional file 1.

During the entire experiment, approximately 100% of the water in each tank was manually changed daily. Water was prepared at the desired temperature before pumping into the tanks. Water quality (dissolved oxygen (DO), pH, nitrate, and ammonia) was monitored thrice weekly using a YSI Pro Plus Multiparameter (YSI Incorporated, Yellow Springs, Ohio, USA), and salinity was measured daily using a seawater refractometer. DO content was > 8.7 mg∙L^− 1^, photoperiod was 12:12-h (light/dark), pH was 7.2 ± 0.7, ammonia level was 0.04 ± 0.02 mg∙L^− 1^, and nitrate was 1.17 ± 0.33 mg∙L^− 1^.

### Experimental design of trial 1

In trial 1, rainbow trout with similar initial weights (94.73 ± 9.70 g) were randomly assigned to nine tanks (186 L; 0.58 m height × 0.65 m diameter). Each treatment was assessed using ten fish per tank (*n* = 3; biomass, 5.09 kg∙m^− 3^, 90 fish in total), following the methodology described by Jenkins et al. [52], and performed in triplicate. The positions of all tanks were randomized based on the assignment of random numbers generated using the RAND function of Microsoft Excel 2019 (Microsoft Corp., Redmond, DC, US). The temperature of dechlorinated freshwater was controlled by a temperature control system (ZKH-WK 2000, Zhongkehai, Qingdao, China), fluctuating within 0.5 °C. The temperature regimes were as follows: water temperature was gradually adjusted from 16 °C (control) to 12.5 and 9 °C at a rate of 2 °C per 12 h, and then kept constant for the rest of the experiment. The fish were subjected to temperature acclimation in freshwater for 28 d. Immediately after acclimation, the salinity of all tanks was increased from 0 ppt to 14 ppt by mixing with seawater (salinity 30 ± 1 ppt) within 60 min, and then further increased from 14 to 30 ppt at a rate of 2 ppt day^− 1^, according to the method described by Ge et al. [26]. Filtered and dechlorinated seawater was drawn from the coastal waters of the Yellow Sea. Water at the desired salinity was prepared in advance and pumped into the tanks. The fish were then stored for 14 days.

### Experimental design of trial 2

In trial 2, juvenile rainbow trout with a similar initial weight (62.28 ± 0.41 g) were randomly allocated to 12 tanks. Each treatment was assessed using ten fish per tank (*n* = 4; biomass, 3.35 kg∙m^− 3^, 120 fish in total), in accordance with the guidelines reported by Jenkins et al. [52], and performed as four replicates. All experimental tanks were randomly positioned as described for trial 1. The temperature of the dechlorinated freshwater was controlled using an AI temperature controller (AI-526P, Yudian Tech, Xiamen, China) with an accuracy of 0.05 °C. The temperature regimes were conducted as follows: water temperature was gradually adjusted from 16.0 to 13.0 °C in 12 h, after which the fish were subjected to three experimental thermal regimes. These regimes are referred to as CT (control), VT2, and VT4. CT mimics a laboratory scenario where the temperature is constant at 13.0 °C, while VT2 and VT4 mimic a natural scenario where the temperature oscillates daily by the same magnitude. In VT2, the fluctuation in temperature is 2.0 °C, changing between 12.0 and 14.0 °C, in a sinusoid manner. VT4 represents a scenario with a larger daily magnitude of fluctuation between 11.0 and 15.0 °C, also in a sinusoid manner. The fish were subjected to temperature acclimation in freshwater for 6 weeks. Immediately after acclimation, the salinity of all tanks was increased from 0 ppt to 15 ppt by mixing with seawater (salinity 30 ± 1 ppt), and then further increased to 30 ppt from 15 ppt at a rate of 3 ppt per day, according to the method described by Ge et al. [11]. Water at the desired salinity was prepared in advance and pumped into the tanks. Fish were then stocked for 3 weeks.

### Sampling procedures

Prior to sampling, fish were fasted for 24 h and euthanized using MS-222 (60 mg∙L^− 1^; Sigma-Aldrich, St. Louis, MO, USA). The sampling order was randomized at each sampling point. For each treatment, the liver and anterior intestine were dissected on ice on the final day in freshwater (FW-28 in trial 1 and FW-42 in trial 2), and on the first (SW-1) and final (SW-14 in trial 1 and SW-21 in trial 2) days after seawater acclimation. Tissues were washed to remove blood and impurities using NaCl (0.9%, w/v), immediately frozen in liquid nitrogen, and stored at − 80 °C for PLFA composition analysis.

### Lipid extraction and PLFA analysis

Using the method described by Folch et al. [53], liver and intestine (0.1 g) samples were placed in 1.9 mL of chloroform/methanol (2:1, v/v) containing 0.01% (w/v) butylated hydroxytoluene (BHT) and homogenized. After extracting the total lipids, the crude extract was thoroughly mixed with 0.5 mL of CaCl_2_ (0.02%, w/v) for protein precipitation. After standing overnight at 4 °C, the mixture was separated into two phases, the upper phase of which was removed and the lower phase dried to a constant weight under a flow of nitrogen to obtain lipids. The extracted lipids were then dissolved in 20 μL petroleum ether (60–90 °C), spotted on one-dimensional thin-layer hybrid silica gel plates (100 mm × 100 mm; Haiyang Company, Qingdao, China), and developed in an N-hexane/ether/acetic (84:15:1, v/v/v) solvent. Phospholipids were visualized using iodine vapor and scratched from silica gel plates. The silica gel powder containing phospholipids was esterified with 2 mL methyl esterification reagent (hydrochloric acid/methanol, 1:5, v/v) at 90 °C for 90 min to obtain fatty acid methyl esters (FAMEs), which were subsequently extracted in 1 mL of N-hexane, dried under a flow of nitrogen, and dissolved in 50 μL of N-hexane.

The FAMEs were injected into a gas chromatograph (GC-2010 plus; Shimadzu, Kyoto, Japan) equipped with an RTX-WAX fused silica capillary column (30 m × 0.25 mm × 0.25 μm, Phenomenex, Torrance, CA, USA) and a flame ionisation detector (FID; GC-2010, Shimadzu, Kyoto, Japan). The temperature setting of the column oven started from an initial temperature of 80 °C for 1 min, and then serially increased as follows: 180 °C at a rate of 8 °C min^− 1^ for 5 min, 220 °C at a rate of 4 °C min^− 1^, 224 °C at a rate of 0.5 °C min^− 1^, and 280 °C at a rate of 4 °C min^− 1^ for 10 min. FAMEs were classified and quantified by comparing retention times and areas of the peak using the 37-FAME Mix standards (Supelco, Bellefonte, Pennsylvania, USA).

### Biometric indices

The indices of PLFA composition (FA-related indices) were calculated according to the following formulae, as previously used by Wallaert et al. [54], Snyder et al. [55], and Cornelius et al. [56]:

$$ {\displaystyle \begin{array}{c}\mathrm{U}\mathrm{I}=\sum \left(\% monoene+2\times \% dienes+3\times \% trienes\dots \right)/100\\ {}\mathrm{U}/\mathrm{S}=\sum \% UFA/\sum \% SFA\\ {}\mathrm{ACL}=\sum \left(14\times \%14C+16\times \%16C+17\times \%17C\dots \right)/100\end{array}} $$

UI: unsaturation index; monoene, dienes, trienes: the number of double bonds of fatty acids; %: weight percentage; U/S: ratio of unsaturated fatty acids to saturated fatty acids; UFA: unsaturated fatty acids; SFA: saturated fatty acids; ACL: average chain length of phospholipid; n-3 PUFA: omega-3 series polyunsaturated fatty acids; n-6 PUFA: omega-6 series polyunsaturated fatty acids; n3/n6: the ratio of n-3 PUFA to n-6 PUFA.

### Statistical analyses

For data processing and analysis, the experimenters were blinded with respect to animal allocation. All experimental data obtained were subjected to statistical analyses without exclusion. In this study, the experimental designs in both trial 1 and trial 2 were completely randomized designs.

The PLFA data obtained in both trials 1 and 2 are presented as percentage compositions of total fatty acids and were arcsine-square root transformed to meet the requirements for normality and homogeneity. The normality and homogeneity of variances were determined using Levene’s test, and statistical significance was assessed using one- and two-way analyses of variance (ANOVA) using the Student–Newman–Keuls multiple range test with the GLM procedures in SAS 9.4 (SAS Institute, Cary, NC, USA).

To simplify the data interpretation, principal component analysis (PCA) and data visualization were performed using GraphPad Prism 9.0.0 (GraphPad Software Inc., San Diego, CA, USA). All data are expressed as the means ± SD and statistical significance was set at *P* < 0.05.

## Supplementary Information

**Additional file 1.** Fatty acid composition of the diet.

**Additional file 2.** Integrated data of PLFA composition in this research.

**Additional file 3 ***P* values of two-way ANOVA for the effects of temperature variations and seawater acclimation on PLFA-related indices in rainbow trout.

## Data Availability

The data that support the findings of this study are available from the corresponding author upon reasonable request.

## Abbreviations

ACL: Average chain length of fatty acid
ANOVA: Analysis of variance
CT: Constant temperature of 13 °C
DO: Dissolved oxygen
FAMEs: Fatty acid methyl esters
FW-28: The 28th day after rearing in freshwater
FW-42: The 42nd day after rearing in freshwater
MUFA: Monounsaturated fatty acid
n3/n6: Ratio of n-3 PUFA to n-6 PUFA
PCA: Principal component analysis
PLFA: Phospholipid fatty acid
PUFA: Polyunsaturated fatty acid
SFA: Saturated fatty acid
SW-1: The first day after seawater acclimation
SW-14: The 14th day after seawater acclimation
SW-21: The 21st day after seawater acclimation
UFA: Unsaturated fatty acid
U/S: Ratio of unsaturated fatty acid to saturated fatty acid
UI: Unsaturation index
VT2: Diel temperature variation of 2 °C
VT4: Diel temperature variation of 4 °C

## References

[1] FAO (2020). FAO Yearbook.

[2] Xu H, Turchini GM, Francis DS, Liang M, Mock TS, Rombenso A (2020). Are fish what they eat? A fatty acid’s perspective. Prog Lipid Res.

[3] Han L, Guo Y, Dong S (2016). Research on establishing a national offshore aquaculture experimental zone eased on the development of the Yellow Sea cold water mass. Pac J Theol.

[4] Dong S (2019). Researching progresses and prospects in large salmonidae farming in cold water mass of Yellow Sea. J Ocean Univ China.

[5] Bjerknes V, Duston J, Knox D, Harmon P (1992). Importance of body size for acclimation of underyearling Atlantic salmon parr (*Salmo salar* L.) to seawater. Aquaculture..

[6] Handeland SO, Imsland AK, Bjornsson BT, Stefansson SO (2013). Long-term effects of photoperiod, temperature and their interaction on growth, gill Na^+^, K^+^-ATPase activity, seawater tolerance and plasma growth-hormone levels in Atlantic salmon *Salmo salar*. J Fish Biol.

[7] Morgan JD, Iwama GK (1991). Effects of salinity on growth, metabolism, and ion regulation in juvenile rainbow and steelhead trout (*Oncorhynchus mykiss*) and fall Chinook salmon (*Oncorhynchus tshawytscha*). Can J Fish Aquat Sci.

[8] Huang M, Zhou Y, Liu C, Davis DA, Li L, Gao Q (2020). Fatty acid composition, osmolality, Na^+^, K^+^-ATPase activity, cortisol content and antioxidant status of rainbow trout (*Oncorhynchus mykiss*) in response to various dietary levels of eicosapentaenoic acid and docosahexaenoic acid. Aquac Res.

[9] Huang M, Zhou Y, Ge J, Agustsson T, Li L, Gao Q (2020). Fatty acid composition and digestive enzyme activities of rainbow trout in response to dietary docosahexaenoic acid (DHA) and eicosapentaenoic acid (EPA) during salinity acclimation. J Ocean Univ China.

[10] Handeland S, Berge Å, Björnsson BT, Lie Ø, Stefansson S (2000). Seawater adaptation by out-of-season Atlantic salmon (*Salmo salar* L.) smolts at different temperatures. Aquaculture..

[11] Ge J, Huang M, Zhou Y, Deng Q, Liu R, Gao Q (2021). Effects of seawater acclimation at constant and diel cyclic temperatures on growth, osmoregulation and branchial phospholipid fatty acid composition in rainbow trout *Oncorhynchus mykiss*. J Comp Physiol B.

[12] Andersen OS, Koeppe RE (2007). Bilayer thickness and membrane protein function: an energetic perspective. Annu Rev Biophys Biomol Struct.

[13] Ernst R, Ejsing CS, Antonny B (2016). Homeoviscous adaptation and the regulation of membrane lipids. J Mol Biol.

[14] Gennis RB (1989). Biomembranes: molecular structure and function.

[15] Liu C, Zhou Y, Dong K, Sun D, Gao Q, Dong S (2018). Differences in fatty acid composition of gill and liver phospholipids between steelhead trout *Oncorhynchus mykiss* and Atlantic salmon *Salmo salar* under declining temperatures. Aquaculture..

[16] Liu C, Ge J, Zhou Y, Thirumurugan R, Gao Q, Dong S (2020). Effects of decreasing temperature on phospholipid fatty acid composition of different tissues and hematology in Atlantic salmon (*Salmo salar*). Aquaculture..

[17] Shivkamat P, Roy R (2005). Regulation of membrane lipid bilayer structure during salinity adaptation: a study with the gill epithelial cell membranes of *Oreochromis niloticus*. Comp Biochem Physiol B.

[18] Hernández-Pérez J, Naderi F, Chivite M, Soengas JL, Míguez JM, López-Patiño MA (2019). Influence of Stress on Liver Circadian Physiology. A Study in Rainbow Trout, *Oncorhynchus mykiss*, as Fish Model. Front Physiol.

[19] Pounder KC, Mitchell JL, Thomson JS, Pottinger TG, Sneddon LU (2018). Physiological and behavioural evaluation of common anaesthesia practices in the rainbow trout. Appl Anim Behav Sci.

[20] Øverli Ø, Winberg S, Pottinger TG (2005). Behavioral and neuroendocrine correlates of selection for stress responsiveness in rainbow trout - a review. Integr Comp Biol.

[21] Kelly AM, Kohler CC (1999). Cold tolerance and fatty acid composition of striped bass, white bass, and their hybrids. N Am J Aquac.

[22] Snyder RJ, Schregel WD, Wei Y (2012). Effects of thermal acclimation on tissue fatty acid composition of freshwater alewives *Alosa pseudoharengus*. Fish Physiol Biochem.

[23] Fadhlaoui M, Couture P (2016). Combined effects of temperature and metal exposure on the fatty acid composition of cell membranes, antioxidant enzyme activities and lipid peroxidation in yellow perch (*Perca flavescens*). Aquat Toxicol.

[24] Bystriansky JS, Ballantyne JS (2007). Gill Na^+^-K^+^-ATPase activity correlates with basolateral membrane lipid composition in seawater- but not freshwater-acclimated Arctic char (*Salvelinus alpinus*). Am J Physiol-Reg I.

[25] Han C, Dong S, Li L, Gao Q, Zhou Y (2021). Assessment of phospholipid fatty acid profiles for discrimination of salmonids cultured in freshwater and seawater. Food Control.

[26] Ge J, Huang M, Zhou Y, Liu C, Han C, Gao Q (2021). Effects of different temperatures on seawater acclimation in rainbow trout *Oncorhynchus mykiss*: osmoregulation and branchial phospholipid fatty acid composition. J Comp Physiol B.

[27] Austreng E, Storebakken T, Åsgård T (1987). Growth rate estimates for cultured Atlantic salmon and rainbow trout. Aquaculture..

[28] Wang M, Lu D (2005). Diurnal and seasonal variation of clear-sky land surface temperature of several representative land surface types in China retrieved by GMS 5. Acta Meteorol Sin.

[29] Donaldson MR, Cooke SJ, Patterson DA, Macdonald JS (2008). Cold shock and fish. J Fish Biol.

[30] Farkas T, Fodor E, Kitajka K, Halver JE (2001). Response of fish membranes to environmental temperature. Aquac Res.

[31] Hazel JR (1984). Effects of temperature on the structure and metabolism of cell membranes in fish. Am J Physiol-Reg I.

[32] Skalli A, Robin JH, Le Bayon N, Le Delliou H, Person-Le RJ (2006). Impact of essential fatty acid deficiency and temperature on tissues’ fatty acid composition of European sea bass (*Dicentrarchus labrax*). Aquaculture..

[33] Leray C, Chapelle S, Duportail G, Florentz A (1984). Changes in fluidity and 22:6(n-3) content in phospholipids of trout intestinal brush-border membrane as related to environmental salinity. BBA-Biomembranes..

[34] Blicher A, Wodzinska K, Fidorra M, Winterhalter M, Heimburg T (2009). The temperature dependence of lipid membrane permeability, its quantized nature, and the influence of anesthetics. Biophys J.

[35] Tocher DR, Bendiksen EÅ, Campbell PJ, Bell JG (2008). The role of phospholipids in nutrition and metabolism of teleost fish. Aquaculture..

[36] Borlongan IG, Benitez LV (1992). Lipid and fatty acid composition of milkfish (*Chanos chanos* Forsskal) grown in freshwater and seawater. Aquaculture..

[37] Genz J, Esbaugh AJ, Grosell M (2011). Intestinal transport following transfer to increased salinity in an anadromous fish (*Oncorhynchus mykiss*). Comp Biochem Phys A.

[38] Sundh H, Kvamme BO, Fridell F, Olsen RE, Ellis T, Taranger GL (2010). Intestinal barrier function of Atlantic salmon (*Salmo salar* L.) post smolts is reduced by common sea cage environments and suggested as a possible physiological welfare indicator. BMC Physiol.

[39] Brijs J, Hennig GW, Gräns A, Dekens E, Axelsson M, Olsson C (2017). Exposure to seawater increases intestinal motility in euryhaline rainbow trout (*Oncorhynchus mykiss*). J Exp Biol.

[40] Edwards SL, Marshall WS (2012). 1 - Principles and Patterns of Osmoregulation and Euryhalinity in Fishes. In:Edited by McCormick SD, Farrell AP, Brauner CJ. Fish Physiol.

[41] Eldridge WH, Sweeney BW, Law JM (2015). Fish growth, physiological stress, and tissue condition in response to rate of temperature change during cool or warm diel thermal cycles. Can J Fish Aquat Sci.

[42] Sims DW, Wearmouth VJ, Southall EJ, Hill JM, Moore P, Rawlinson K (2006). Hunt warm, rest cool: bioenergetic strategy underlying diel vertical migration of a benthic shark. J Anim Ecol.

[43] Thomas RE, Gharrett JA, Carls MG, Rice SD, Moles A, Korn S (1986). Effects of fluctuating temperature on mortality, stress, and energy reserves of juvenile coho salmon. Trans Am Fish Soc.

[44] Lushchak VI (2011). Environmentally induced oxidative stress in aquatic animals. Aquat Toxicol.

[45] Martínez-Álvarez RM, Morales AE, Sanz A (2005). Antioxidant defenses in fish: biotic and abiotic factors. Rev Fish Biol Fish.

[46] Tunnah L, Currie S, Maccormack TJ (2017). Do prior diel thermal cycles influence the physiological response of Atlantic salmon (*Salmo salar*) to subsequent heat stress?. Can J Fish Aquat Sci.

[47] Hokanson KE, Kleiner CF, Thorslund TW (1977). Effects of constant temperatures and diel temperature fluctuations on specific growth and mortality rates and yield of juvenile rainbow trout, *Salmo gairdneri*. J Fish Board Can.

[48] McCormick SD (2001). Endocrine control of osmoregulation in teleost fish. Am Zool.

[49] Johannsson A, Smith GA, Metcalfe JC (1981). The effect of bilayer thickness on the activity of (Na^+^ + K^+^)-ATPase. BBA-Biomembranes..

[50] Vorobyov I, Olson Timothy E, Kim Jung H, Koeppe Roger E, Andersen Olaf S, Allen TW (2014). Ion-induced defect permeation of lipid membranes. Biophys J.

[51] Percie du Sert N, Ahluwalia A, Alam S, Avey MT, Baker M, Browne WJ (2020). Reporting animal research: Explanation and elaboration for the ARRIVE guidelines 2.0. PLoS Biol.

[52] Jenkins JA, Bart HL, Bowker JD, Bowser PR, MacMillan JR, Nickum JG (2014). Guidelines for use of fishes in research – revised and expanded, 2014. Fisheries..

[53] Folch J, Lees M, Stanley GS (1957). A simple method for the isolation and purification of total lipides from animal tissues. J Biol Chem.

[54] Wallaert C, Babin PJ (1994). Thermal adaptation affects the fatty acid composition of plasma phospholipids in trout. Lipids..

[55] Snyder RJ, Hennessey TM (2003). Cold tolerance and homeoviscous adaptation in freshwater alewives *Alosa pseudoharengus*. Fish Physiol Biochem.

[56] Cornelius F, Turner N, Christensen HRZ (2003). Modulation of Na,K-ATPase by Phospholipids and Cholesterol. II Steady-State and Presteady-State Kinetics. Biochemistry.

