# Supplementary material for: Effects of constant and diel cyclic temperatures on the liver and intestinal phospholipid fatty acid composition in rainbow trout Oncorhynchus mykiss during seawater acclimation
Source: BMC Zool. 2021 Jun 21;6:21. doi: 10.1186/s40850-021-00086-6 (PMC10127026; doi:10.1186/s40850-021-00086-6)
Supplement: Supplementary file 1 — Additional file 1. Fatty acid composition of the diet. [file 40850_2021_86_MOESM1_ESM.docx]

**Effects of constant and diel cyclic temperatures on the liver and intestinal phospholipid fatty acid composition in rainbow trout *Oncorhynchus mykiss* during seawater acclimation**

Jian Ge^1^, Yangen Zhou^1, 2^*, Ming Huang^1^, Qinfeng Gao^1, 2^, Yunwei Dong^1, 2^, Shuanglin Dong^1, 2^

^1^Key Laboratory of Mariculture, Ministry of Education, Ocean University of China, Qingdao, Shandong Province, China

^2^Function Laboratory for Marine Fisheries Science and Food Production Processes, Qingdao National Laboratory for Marine Science and Technology, Qingdao, Shandong Province 266235, China

*Corresponding Author: Yangen Zhou, Key Laboratory of Mariculture (Ocean University of China), Qingdao 266100, China. Tel: +86 532 8203 1590; e-mail: [zhouyg@ouc.edu.cn](mailto:zhouyg@ouc.edu.cn)

**Supplementary Table S1** Fatty acid composition of the diet (%).

| SFA | Trial 1 | Trial 2 | MUFA | Trial 1 | Trial 2 | PUFA | Trial 1 | Trial 2 |
| --- | --- | --- | --- | --- | --- | --- | --- | --- |
| 12:0 | 0.20 | 0.08 | 14:1n5 | 0.09 | 0.02 | 18:2n6 | 32.20 | 23.92 |
| 13:0 | 0.04 | 0.03 | 15:1n5 | 0.06 | 0.02 | 18:3n3 | 3.72 | 2.30 |
| 14:0 | 2.44 | 3.70 | 16:1n7 | 2.48 | 4.61 | 18:3n6 | 0.09 | 0.12 |
| 15:0 | 0.27 | 0.46 | 17:1n7 | 0.16 | 0.05 | 20:3n3 | 4.16 | 1.32 |
| 16:0 | 16.03 | 18.88 | 18:1n9 | 20.21 | 18.84 | 20:3n6 | 0.02 | 0.10 |
| 17:0 | 0.54 | 0.43 | 20:1n9 | 0.15 | 0.22 | 20:4n6 | 0.60 | 0.81 |
| 18:0 | 4.46 | 4.20 | 22:1n9 | 0.09 | 0.12 | 20:5n3 | 3.83 | 6.92 |
| 20:0 | 0.56 | 0.32 | 24:1n9 | 0.54 | 0.10 | 22:2n6 | 0.06 | 0.16 |
| 21:0 | 0.06 | 0.05 | ƩMUFA | 23.78 | 23.98 | 22:6n3 | 6.21 | 11.67 |
| 22:0 | 0.47 | 0.24 |  |  |  | ƩPUFA | 50.89 | 47.32 |
| 23:0 | 0.05 | 0.05 |  |  |  |  |  |  |
| 24:0 | 0.21 | 0.25 |  |  |  |  |  |  |
| ƩSFA | 25.33 | 28.70 |  |  |  |  |  |  |

Note: SFA: saturated fatty acid, MUFA: monounsaturated fatty acid, PUFA: polyunsaturated fatty acid.
