# Supplementary material for: Effects of constant and diel cyclic temperatures on the liver and intestinal phospholipid fatty acid composition in rainbow trout Oncorhynchus mykiss during seawater acclimation
Source: BMC Zool. 2021 Jun 21;6:21. doi: 10.1186/s40850-021-00086-6 (PMC10127026; doi:10.1186/s40850-021-00086-6)
Supplement: Supplementary file 2 — Additional file 2. Integrated data of PLFA composition in this research. [file 40850_2021_86_MOESM2_ESM.docx]

**Supplementary Table S2** Liver phospholipid fatty acid composition of rainbow trout in different constant temperatures during seawater acclimation.

| SP | FW-28 | | | SW-1 | | | SW-14 | | |
| --- | --- | --- | --- | --- | --- | --- | --- | --- | --- |
| Temp. | 9 °C | 12.5 °C | 16 °C | 9 °C | 12.5 °C | 16 °C | 9 °C | 12.5 °C | 16 °C |
| Saturated fatty acid | | |  |  |  |  |  |  |  |
| C14:0 | 1.11±0.02^Ab^ | 1.11±0.03^A^ | 0.82±0.35^B^ | 1.38±0.07^Aa^ | 0.83±0.31^B^ | 0.97±0.22^B^ | 1.04±0.42^b^ | 1.25±0.15 | 1.03±0.43 |
| C16:0 | 17.09±1.21^Ba^ | 17.39±1.34^B^ | 18.95±1.19^Aa^ | 17.40±1.25^Aa^ | 17.55±1.18^A^ | 14.80±1.28^Bb^ | 15.53±1.29^Bb^ | 18.78±2.40^A^ | 14.56±1.34^Bb^ |
| C17:0 | 0.44±0.05 | 0.42±0.03 | 0.41±0.02 | 0.37±0.14 | 0.35±0.23 | 0.60±0.21 | 0.57±0.22 | 0.52±0.334 | 0.49±0.11 |
| C18:0 | 9.45±0.27^Ab^ | 7.40±0.14^Bb^ | 9.30±0.26^A^ | 11.29±1.06^Aa^ | 10.24±0.82^Ba^ | 9.21±0.15^C^ | 9.18±0.83^Ab^ | 9.25±0.88^Aa^ | 8.65±1.36^B^ |
| C20:0 | 0.28±0.13 | 0.25±0.09 | 0.35±0.06^a^ | 0.20±0.12 | 0.19±0.11 | 0.24±0.07^ab^ | 0.22±0.12 | 0.29±0.24 | 0.18±0.02^b^ |
| C22:0 | 0.05±0.02^b^ | 0.08±0.02 | 0.09±0.03^b^ | 0.18±0.08^Aa^ | 0.05±0.02^B^ | 0.06±0.01^Bb^ | 0.05±0.02^Bb^ | 0.08±0.02^B^ | 0.23±0.05^Aa^ |
| C23:0 | 0.25±0.12^b^ | 0.52±0.22^a^ | 0.39±0.12^b^ | 0.63±0.13^a^ | 0.54±0.22^a^ | 0.70±0.29^a^ | 0.05±0.02^Bc^ | 0.06±0.02^Bb^ | 0.23±0.11^Ab^ |
| C24:0 | 0.33±0.13^Ac^ | 0.41±0.16^AB^ | 0.60±0.18^Ab^ | 0.65±0.24^Bb^ | 0.63±0.22^B^ | 1.16±0.11^Aa^ | 1.08±0.32^Aa^ | 0.51±0.25^B^ | 1.15±0.43^Aa^ |
| ƩSFA | 29.00±0.26^Bb^ | 27.57±0.31^Cb^ | 30.9±0.36^Aa^ | 32.11±0.35^Aa^ | 30.38±0.32^Ba^ | 27.75±0.14^Cb^ | 27.72±1.36^Bc^ | 30.73±2.46^Aa^ | 26.51±1.04^Bb^ |
| Monounsaturated fatty acid | | |  |  |  |  |  |  |  |
| C14:1n5 | 0.73±0.11 | 0.60±0.31^b^ | 0.55±0.23^b^ | 0.54±0.32 | 0.62±0.31^b^ | 1.01±0.22^a^ | 1.09±0.41 | 1.33±0.81^a^ | 1.05±0.42^a^ |
| C16:1n5 | 1.07±0.10^C^ | 2.18±0.12^Aa^ | 1.96±0.05^Bb^ | 1.21±0.52 | 1.71±0.42^b^ | 1.35±0.45^c^ | 1.24±0.62^B^ | 1.86±0.41^Bb^ | 3.29±0.98^Aa^ |
| C17:1n7 | 0.25±0.11^a^ | 0.20±0.13 | 0.14±0.08 | 0.13±0.05^b^ | 0.15±0.04 | 0.20±0.12 | 0.26±0.02^a^ | 0.17±0.05 | 0.21±0.11 |
| C18:1n9 | 7.23±0.12^C^ | 11.55±0.17^Aa^ | 9.18±0.2^Bb^ | 7.33±1.12^B^ | 8.51±1.13^ABb^ | 9.79±1.25^Ab^ | 7.68±1.06^B^ | 7.48±1.22^Bc^ | 13.95±2.52^Aa^ |
| C20:1n9 | 0.28±0.05 | 0.21±0.08 | 0.30±0.06 | 0.24±0.05 | 0.22±0.07 | 0.25±0.03 | 0.26±0.02 | 0.30±0.12 | 0.25±0.09 |
| C22:1n9 | 0.44±0.08^a^ | 0.31±0.13 | 0.49±0.14 | 0.25±0.07^b^ | 0.23±0.08 | 0.33±0.04 | 0.05±0.02^Bc^ | 0.34±0.14^A^ | 0.41±0.09^A^ |
| C24:1n9 | 3.05±0.25^Aa^ | 2.55±0.12^B^ | 3.16±0.16^A^ | 2.57±0.19^Bb^ | 2.44±0.27^B^ | 3.24±0.29^A^ | 3.39±1.08^Aab^ | 2.67±0.76^AB^ | 2.41±0.48^B^ |
| ƩMUFA | 13.04±0.87^C^ | 17.59±1.28^Aa^ | 15.78±1.25^Bb^ | 12.26±1.10^B^ | 13.87±1.22^Bb^ | 16.18±2.23^Ab^ | 13.98±1.16^B^ | 14.16±2.26^Bb^ | 21.57±2.58^Aa^ |
| Polyunsaturated fatty acid | | |  |  |  |  |  |  |  |
| C18:2n6 | 4.57±0.13^Ba^ | 5.25±0.03^Aa^ | 4.17±0.18^Cb^ | 3.61±0.80^b^ | 3.43±0.74^c^ | 4.24±0.93^ab^ | 4.58±1.27^ab^ | 4.56±0.86^b^ | 5.52±1.32^a^ |
| C18:3n3 | 2.01±0.22^a^ | 2.02±0.09^a^ | 2.07±0.08 | 1.71±0.17^Bb^ | 1.64±0.23^Bc^ | 1.94±0.42^A^ | 2.11±0.42^Aa^ | 1.87±0.19^Bb^ | 2.03±0.19^A^ |
| C18:3n6 | 0.23±0.02 | 0.19±0.02 | 0.21±0.01 | 0.17±0.09 | 0.11±0.04 | 0.14±0.02 | 0.18±0.02 | 0.15±0.04 | 0.14±0.01 |
| C20:2n6 | 1.85±0.41^Aa^ | 1.09±0.26^AB^ | 0.82±0.25^B^ | 0.95±0.13^ABc^ | 0.87±0.21^B^ | 1.44±0.34^A^ | 1.52±0.47^b^ | 1.35±0.53 | 1.51±0.21 |
| C20:3n3 | 2.07±0.23^Ab^ | 1.26±0.45^Bb^ | 1.19±0.65^B^ | 1.23±0.23^c^ | 2.04±0.44^a^ | 1.52±0.50 | 2.19±0.56^Aa^ | 1.57±0.16^Bb^ | 1.24±0.12^C^ |
| C20:3n6 | 1.06±0.31^a^ | 1.60±0.16^a^ | 1.83±0.47^a^ | 0.66±0.12^b^ | 0.93±0.13^b^ | 0.83±0.26^b^ | 0.62±0.12^Bb^ | 1.05±0.27^Ab^ | 0.64±0.23^Bb^ |
| C20:4n6 | 5.30±0.34^Aa^ | 4.26±0.24^Ba^ | 2.72±0.22^Cb^ | 3.73±0.16^Bc^ | 3.59±0.34^Bb^ | 4.76±0.71^Aa^ | 4.61±0.31^b^ | 4.42±0.36^a^ | 4.02±0.72^a^ |
| C20:5n3 | 4.36±0.54^b^ | 4.14±0.65 | 4.39±0.29 | 5.40±0.63^Aa^ | 4.61±0.32^B^ | 4.63±0.76^B^ | 5.87±0.27^Aa^ | 4.71±0.51^B^ | 4.98±0.61^B^ |
| C22:2n6 | 0.20±0.06^b^ | 0.17±0.09^b^ | 0.11±0.04^b^ | 0.08±0.04^Bc^ | 0.45±0.13^Aa^ | 0.34±0.12^Aa^ | 0.30±0.04^Aa^ | 0.09±0.02^Cb^ | 0.21±0.02^Bab^ |
| C22:6n3 | 36.31±0.28^Ab^ | 34.85±0.48^Cb^ | 35.81±0.25^Ba^ | 38.09±2.29^Aa^ | 38.07±2.41^Aa^ | 36.23±1.15^Ba^ | 36.31±2.26^Ab^ | 35.34±2.30^Ab^ | 31.67±1.57^Bb^ |
| ƩPUFA | 57.96±2.91^Aa^ | 54.84±1.58^B^ | 53.32±1.12^Cb^ | 55.63±2.25^b^ | 55.75±3.25 | 56.07±5.11^a^ | 58.30±2.27^Aa^ | 55.11±2.41^B^ | 51.92±2.62^Cb^ |

Note: Values are means of 3 replications. Different lowercase letters indicate significant differences (*P* < 0.05) among different treatments at the same time, and different capital letters indicate significant differences (*P* < 0.05) at different times at the same treatment based on one-way ANOVA analysis followed by the Student-Newman-Keuls (SNK) test. FW-28: end of growth trial. SW-1: one day after salinity reached 30. SW-4: four days after salinity reached 30. SW-7: seven days after salinity reached 30. SW-14: fourteen days after salinity reached 30.

**Supplementary Table S3** Intestine phospholipid fatty acid composition of rainbow trout in different constant temperatures during seawater acclimation.

| SP | FW-28 | | | SW-1 | | | SW-14 | | |
| --- | --- | --- | --- | --- | --- | --- | --- | --- | --- |
| Temperature | 9 °C | 12.5 °C | 16 °C | 9 °C | 12.5 °C | 16 °C | 9 °C | 12.5 °C | 16 °C |
| Saturated fatty acid | |  |  |  |  |  |  |  |  |
| C14:0 | 2.73±0.19^B^ | 2.26±0.17^C^ | 3.32±0.25^Aa^ | 2.48±0.81 | 1.67±0.61 | 2.20±0.68^ab^ | 2.50±0.41 | 2.49±0.71 | 1.63±0.64^b^ |
| C16:0 | 22.31±1.24 | 22.06±1.38 | 23.99±1.57^a^ | 22.37±1.34 | 21.55±1.15 | 23.58±1.08^a^ | 21.16±1.06^B^ | 23.10±0.81^A^ | 21.38±0.86^Bb^ |
| C17:0 | 0.78±0.21^B^ | 0.49±0.11^Bb^ | 1.25±0.22^Aa^ | 0.81±0.14^A^ | 0.75±0.07^ABa^ | 0.58±0.08^Bb^ | 0.62±0.02 | 0.54±0.01^b^ | 0.67±0.01^b^ |
| C18:0 | 10.27±0.73^AB^ | 11.51±0.56^A^ | 10.22±0.68^Bb^ | 9.29±0.92^B^ | 11.69±0.66^A^ | 11.44±0.63^Aab^ | 10.45±1.02 | 11.23±0.99 | 12.57±1.11^a^ |
| C20:0 | 0.29±0.02 | 0.32±0.03 | 0.45±0.11^a^ | 0.25±0.04 | 0.22±0.06 | 0.31±0.04^ab^ | 0.17±0.03 | 0.26±0.08 | 0.17±0.04^b^ |
| C22:0 | 0.18±0.03^A^ | 0.10±0.02^Ba^ | 0.11±0.03^B^ | 0.14±0.05 | 0.16±0.06^a^ | 0.18±0.04 | 0.11±0.04 | 0.05±0.01^b^ | 0.16±0.02 |
| C23:0 | 0.12±0.04^AB^ | 0.07±0.02^B^ | 0.13±0.03^A^ | 0.13±0.04^A^ | 0.04±0.02^B^ | 0.11±0.07^A^ | 0.12±0.04 | 0.07±0.02 | 0.11±0.03 |
| C24:0 | 1.04±0.11^B^ | 0.76±0.07^C^ | 2.91±0.71^Aa^ | 1.05±0.42 | 0.96±0.33 | 1.19±0.22^b^ | 1.54±0.52 | 0.68±0.41 | 1.16±0.71^b^ |
| ƩSFA | 37.71±1.23^B^ | 37.59±1.29^B^ | 42.38±1.44^Aa^ | 36.53±1.03^B^ | 37.04±0.78^B^ | 39.60±1.24^Ab^ | 36.66±1.76 | 38.42±1.16 | 37.84±1.33^c^ |
| Monounsaturated fatty acid | |  |  |  |  |  |  |  |  |
| C14:1n5 | 1.64±0.13^Ba^ | 1.06±0.11^C^ | 2.93±0.72^Aa^ | 0.86±0.05^Cb^ | 1.19±0.04^B^ | 1.61±0.16^Ab^ | 0.93±0.33^b^ | 1.05±0.52 | 0.62±0.11^c^ |
| C16:1n5 | 1.86±0.41 | 1.51±0.32 | 1.51±0.31 | 2.40±0.91^A^ | 1.26±0.63^B^ | 1.39±0.79^B^ | 2.13±0.31^A^ | 1.72±0.12^AB^ | 1.38±0.07^B^ |
| C17:1n7 | 0.53±0.04^a^ | 0.47±0.03 | 0.45±0.08 | 0.29±0.08^Bb^ | 0.45±0.05^A^ | 0.19±0.06^B^ | 0.34±0.05^b^ | 0.40±0.04 | 0.34±0.07 |
| C18:1n9 | 10.85±0.88^a^ | 9.39±0.76^a^ | 10.21±0.92^a^ | 8.20±0.85^Bb^ | 10.68±0.62^Aa^ | 9.47±1.04^ABab^ | 8.32±0.72^b^ | 7.42±0.71^b^ | 8.86±0.83^b^ |
| C20:1n9 | 0.23±0.02^b^ | 0.22±0.02 | 0.35±0.03^a^ | 0.21±0.06^b^ | 0.17±0.03 | 0.19±0.04^b^ | 0.35±0.04^a^ | 0.23±0.03 | 0.27±0.06^ab^ |
| C22:1n9 | 0.60±0.21 | 0.54±0.11^b^ | 0.85±0.14^a^ | 0.71±0.02 | 0.77±0.05^a^ | 0.64±0.04^b^ | 0.52±0.05 | 0.57±0.07^b^ | 0.52±0.13^b^ |
| C24:1 | 2.49±0.55 | 3.22±0.51^a^ | 3.24±0.44^a^ | 3.01±0.63 | 3.49±0.97^a^ | 2.53±0.71^ab^ | 2.39±0.22^A^ | 2.55±0.34^Ab^ | 1.63±0.17^Bb^ |
| ƩMUFA | 18.19±1.07^ABa^ | 16.42±0.96^Ba^ | 19.54±0.71^Aa^ | 15.67±1.07^Bb^ | 18.02±1.17^Aa^ | 16.01±0.91^Bb^ | 14.98±1.31^b^ | 13.93±1.12^b^ | 13.62±0.98^c^ |
| Polyunsaturated fatty acid | |  |  |  |  |  |  |  |  |
| C18:2n6 | 3.83±0.85^Bb^ | 5.52±0.67^Aa^ | 4.96±0.92^Ab^ | 5.07±0.84^a^ | 4.50±0.73^ab^ | 4.19±0.93^b^ | 3.93±0.33^b^ | 3.75±0.66^b^ | 7.01±0.92^a^ |
| C18:3n3 | 2.75±0.61^AB^ | 2.30±0.56^B^ | 3.80±0.81^A^ | 2.63±0.51 | 2.85±0.67 | 3.12±0.81 | 2.82±0.86 | 3.30±0.64 | 2.50±0.73 |
| C18.3n6 | 0.34±0.09^a^ | 0.22±0.07 | 0.43±0.23 | 0.15±0.04^b^ | 0.16±0.04 | 0.18±0.03 | 0.12±0.04^b^ | 0.15±0.03 | 0.24±0.06 |
| C20:2n6 | 0.79±0.22 | 0.70±0.03^a^ | 0.78±0.12 | 0.67±0.06 | 0.72±0.04^a^ | 0.72±0.09 | 0.79±0.08 | 0.56±0.03^b^ | 0.74±0.05 |
| C20:3n3 | 1.01±0.42 | 1.11±0.41 | 0.97±0.25 | 0.75±0.04^B^ | 1.36±0.16^A^ | 0.82±0.09^B^ | 1.17±0.75 | 1.05±0.43 | 0.70±0.12 |
| C20:3n6 | 0.55±0.12 | 0.67±0.13 | 0.73±0.14^a^ | 0.56±0.07^A^ | 0.47±0.11^A^ | 0.31±0.04^Bb^ | 0.45±0.07 | 0.64±0.03 | 0.53±0.06^a^ |
| C20:4n6 | 2.49±0.76 | 2.45±0.64 | 2.61±0.83 | 3.04±1.07 | 2.60±1.04 | 2.30±0.96 | 2.94±0.84 | 2.77±0.91 | 2.29±0.64 |
| C20:5n3 | 1.76±0.53^Bb^ | 2.94±0.62^Aa^ | 2.17±0.72^AB^ | 2.90±0.76^ab^ | 2.39±0.43^b^ | 2.81±0.72 | 3.70±0.73^a^ | 3.70±0.85^a^ | 2.74±0.64 |
| C22:2n6 | 0.33±0.03^a^ | 0.18±0.03^b^ | 0.38±0.04^a^ | 0.15±0.07^Bb^ | 0.31±0.06^Aa^ | 0.25±0.07^Aab^ | 0.16±0.05^b^ | 0.23±0.07^ab^ | 0.19±0.05^b^ |
| C22:6n3 | 30.24±1.22^Ab^ | 29.90±2.09^A^ | 21.25±0.53^Bb^ | 31.89±1.76^ab^ | 29.58±0.89 | 29.71±1.07^a^ | 32.28±2.19^a^ | 31.50±1.15 | 31.61±1.75^a^ |
| ƩPUFA | 44.09±2.06^Ab^ | 45.99±2.36^Aab^ | 38.07±1.68^Bc^ | 47.80±1.49^Aa^ | 44.94±1.34^Bb^ | 44.39±1.17^Bb^ | 48.35±1.13^a^ | 47.65±2.07^a^ | 48.54±1.25^a^ |

Note: Same as Supplementary Table S2.

**Supplementary Table** **S4** Phospholipid fatty acid composition (%) of the liver in rainbow trout at different sampling points at diel cyclic temperatures.

| SP | FW-42 | | | SW-1 | | | SW-21 | | |
| --- | --- | --- | --- | --- | --- | --- | --- | --- | --- |
| Temperature | CT | VT2 | VT4 | CT | VT2 | VT4 | CT | VT2 | VT4 |
| Saturated fatty acids | |  |  |  |  |  |  |  |  |
| C14:0 | 0.96±0.04^b^ | 1.00±0.06^b^ | 0.97±0.05^b^ | 1.18±0.07^a^ | 1.16±0.11^a^ | 1.13±0.10^a^ | 1.07±0.11^ab^ | 1.19±0.06^a^ | 1.11±0.06^a^ |
| C16:0 | 17.26±1.00^AB^ | 15.98±0.71^Bc^ | 17.90±0.68^Aa^ | 16.95±1.03 | 17.40±0.96^b^ | 16.50±0.69^b^ | 18.41±0.99 | 18.77±0.51^a^ | 18.26±0.54^a^ |
| C17:0 | 0.61±0.15^ab^ | 0.53±0.13 | 0.71±0.19^a^ | 0.44±0.15^ABb^ | 0.57±0.15^A^ | 0.26±0.12^Bb^ | 0.74±0.15^a^ | 0.61±0.23 | 0.67±0.07^a^ |
| C18:0 | 6.23±0.46^b^ | 6.21±0.34^b^ | 6.27±0.62^c^ | 7.66±1.11^a^ | 7.63±0.44^a^ | 7.35±0.41^b^ | 8.09±0.67^a^ | 7.70±0.56^a^ | 8.20±0.47^a^ |
| C20:0 | 0.21±0.12 | 0.19±0.04^a^ | 0.19±0.05 | 0.13±0.03 | 0.13±0.01^ab^ | 0.14±0.03 | 0.15±0.05 | 0.09±0.06^b^ | 0.15±0.04 |
| C22:0 | 0.12±0.08 | 0.09±0.03 | 0.16±0.07 | 0.08±0.02 | 0.12±0.10 | 0.08±0.01 | 0.10±0.03 | 0.09±0.05 | 0.08±0.03 |
| C24:0 | 0.52±0.05^Bb^ | 0.94±0.27^A^ | 0.79±0.22^ABa^ | 0.76±0.16^a^ | 0.82±0.39 | 0.38±0.06^b^ | 0.63±0.12^Aab^ | 0.42±0.10^B^ | 0.48±0.07^ABb^ |
| ƩSFA | 25.92±1.37^b^ | 24.94±1.05^b^ | 27.00±0.85^b^ | 27.20±1.53^ab^ | 27.82±0.95^a^ | 25.84±1.07^b^ | 29.19±1.45^a^ | 28.87±0.26^a^ | 28.96±0.96^a^ |
| Monounsaturated fatty acids | |  |  |  |  |  |  |  |  |
| C14:1n5 | 0.79±0.25^b^ | 0.78±0.21 | 0.98±0.11^b^ | 0.60±0.17^b^ | 0.92±0.29 | 0.59±0.14^c^ | 1.42±0.27^a^ | 1.01±0.23 | 1.28±0.17^a^ |
| C16:1n5 | 2.61±0.46^a^ | 2.54±0.22^a^ | 2.70±0.35^a^ | 1.42±0.17^b^ | 2.04±0.64^ab^ | 2.14±0.50^ab^ | 1.23±0.32^b^ | 1.34±0.33^b^ | 1.59±0.13^b^ |
| C17:1n7 | 0.23±0.04 | 0.19±0.03 | 0.20±0.02 | 0.23±0.01^A^ | 0.20±0.03^A^ | 0.17±0.02^B^ | 0.19±0.03 | 0.22±0.02 | 0.21±0.04 |
| C18:1n9 | 2.01±0.24^a^ | 2.09±0.14^a^ | 2.11±0.22^a^ | 1.51±0.04^Bb^ | 1.68±0.19^ABb^ | 1.86±0.16^Aa^ | 1.60±0.16^b^ | 1.55±0.28^b^ | 1.56±0.15^b^ |
| C20:1n9 | 0.20±0.02 | 0.17±0.03 | 0.15±0.04 | 0.18±0.05 | 0.16±0.01 | 0.20±0.01 | 0.18±0.06 | 0.13±0.05 | 0.17±0.01 |
| C22:1n9 | 0.08±0.07 | 0.05±0.02 | 0.08±0.04 | 0.06±0.01 | 0.05±0.03 | 0.05±0.01 | 0.06±0.01 | 0.06±0.03 | 0.04±0.01 |
| C24:1n9 | 2.81±0.33 | 2.91±0.07 | 2.62±0.14^b^ | 2.88±0.10 | 2.75±0.25 | 2.71±0.24^b^ | 3.18±0.16 | 3.01±0.29 | 3.19±0.13^a^ |
| ƩMUFA | 8.72±0.70^a^ | 8.72±0.58^a^ | 8.84±0.35^a^ | 6.88±0.23^Bb^ | 7.80±0.40^Ab^ | 7.72±0.41^Ab^ | 7.88±0.62^a^ | 7.32±0.52^b^ | 8.04±0.44^b^ |
| Polyunsaturated fatty acids | |  |  |  |  |  |  |  |  |
| C18:2n6 | 12.89±0.96^a^ | 12.86±0.7^a^ | 12.92±0.56^a^ | 10.22±0.39^Bb^ | 12.00±0.85^Aa^ | 13.23±0.95^Aa^ | 9.18±1.03^b^ | 8.89±1.24^b^ | 9.58±0.80^b^ |
| C18:3n3 | 0.10±0.02 | 0.10±0.03 | 0.10±0.03^ab^ | 0.15±0.03^A^ | 0.10±0.03^B^ | 0.08±0.01^Bb^ | 0.14±0.02 | 0.14±0.05 | 0.13±0.00^a^ |
| C18:3n6 | 0.16±0.03 | 0.16±0.07 | 0.13±0.02 | 0.13±0.02 | 0.14±0.05 | 0.15±0.03 | 0.15±0.06 | 0.13±0.04 | 0.10±0.02 |
| C20:2n6 | 1.26±0.27^ABa^ | 1.48±0.11^Aa^ | 1.12±0.09^B^ | 1.18±0.21^a^ | 1.21±0.23^ab^ | 1.36±0.16 | 0.79±0.23^b^ | 0.97±0.24^b^ | 1.18±0.20 |
| C20:3n3 | 0.91±0.14^b^ | 0.84±0.12^b^ | 0.84±0.10^b^ | 1.33±0.15^Aa^ | 0.96±0.22^Bb^ | 0.94±0.12^Bb^ | 1.24±0.18^a^ | 1.28±0.13^a^ | 1.21±0.08^a^ |
| C20:3n6 | 1.93±0.26^A^ | 1.50±0.23^Bab^ | 1.29±0.15^Bb^ | 2.03±0.41 | 1.83±0.22^a^ | 2.06±0.18^a^ | 1.82±0.28^A^ | 1.14±0.48^Bb^ | 1.54±0.20^ABb^ |
| C20:4n6 | 2.92±0.72^Ab^ | 2.78±0.11^Ac^ | 1.85±0.20^Bc^ | 3.37±0.26^b^ | 3.53±0.41^b^ | 3.06±0.69^b^ | 5.19±0.70^a^ | 4.93±0.56^a^ | 4.63±0.34^a^ |
| C20:5n3 | 2.69±0.59^b^ | 2.52±0.20 | 2.12±0.24^b^ | 3.01±0.31^ab^ | 2.90±0.11 | 2.80±0.24^a^ | 3.64±0.33^Aa^ | 2.60±0.37^B^ | 2.92±0.20^Ba^ |
| C22:2n6 | 0.34±0.06 | 0.33±0.08 | 0.38±0.12 | 0.23±0.07 | 0.37±0.08 | 0.28±0.08 | 0.23±0.06 | 0.29±0.09 | 0.30±0.07 |
| C22:6n3 | 42.16±1.47^b^ | 43.78±1.29^a^ | 43.4±0.87^a^ | 44.29±0.57^Aa^ | 41.33±0.52^Cb^ | 42.47±0.83^Bab^ | 40.54±1.21^Bb^ | 43.45±1.09^Aa^ | 41.39±0.77^Bb^ |
| ƩPUFA | 65.36±1.88 | 66.34±1.62^a^ | 64.16±1.07^b^ | 65.92±1.58 | 64.38±1.23^ab^ | 66.44±1.29^a^ | 62.93±1.67 | 63.81±0.65^b^ | 63.00±0.94^b^ |

Note: Values represent the mean ± S.D. (n = 4). Different lowercase letters indicate significant differences (*P* < 0.05) among different treatments at the same time, and different capital letters indicate significant differences (*P* < 0.05) at different times at the same treatment based on one-way ANOVA analysis followed by the Student-Newman-Keuls (SNK) test. FW-42: end of growth trial. SW-1: one day after salinity reached 30. SW-21: 21 days after salinity reached 30. SFA: saturated fatty acid, MUFA: monounsaturated fatty acid, PUFA: polyunsaturated fatty acid.

**Supplementary Table S5** Phospholipid fatty acid composition (%) of the intestine in rainbow trout at different sampling points at diel cyclic temperatures.

| SP | FW-42 | | | SW-1 | | | SW-21 | | |
| --- | --- | --- | --- | --- | --- | --- | --- | --- | --- |
| Temperature | CT | VT2 | VT4 | CT | VT2 | VT4 | CT | VT2 | VT4 |
| Saturated fatty acids | |  |  |  |  |  |  |  |  |
| C14:0 | 1.27±0.15^b^ | 1.14±0.23^b^ | 1.02±0.12^c^ | 1.28±0.10^b^ | 1.20±0.10^b^ | 1.25±0.14^b^ | 1.50±0.10^a^ | 1.72±0.22^a^ | 1.67±0.09^a^ |
| C16:0 | 19.84±1.00^ABa^ | 21.32±0.49^A^ | 18.76±1.37^B^ | 16.96±0.65^Bb^ | 20.7±0.95^A^ | 20.46±0.56^A^ | 20.95±0.58^a^ | 21.37±0.96 | 20.20±0.92 |
| C17:0 | 0.62±0.07 | 0.73±0.20 | 0.63±0.09 | 0.82±0.21 | 0.68±0.09 | 1.01±0.31 | 0.88±0.15 | 1.11±0.36 | 0.85±0.11 |
| C18:0 | 11.07±0.56^Aa^ | 10.36±1.14^A^ | 8.69±0.54^Bc^ | 9.19±0.85^Bb^ | 10.41±0.65^B^ | 12.59±1.50^Aa^ | 10.67±0.59^a^ | 9.81±0.67 | 10.49±1.03^b^ |
| C20:0 | 0.27±0.04 | 0.38±0.10^a^ | 0.33±0.05^a^ | 0.26±0.06^AB^ | 0.29±0.03^Aab^ | 0.21±0.02^Bb^ | 0.22±0.04 | 0.23±0.03^b^ | 0.22±0.04^b^ |
| C22:0 | 0.11±0.03 | 0.14±0.04 | 0.13±0.06 | 0.15±0.04 | 0.10±0.03 | 0.14±0.05 | 0.11±0.04 | 0.19±0.08 | 0.12±0.01 |
| C24:0 | 0.77±0.19^B^ | 1.20±0.29^Aa^ | 0.81±0.15^B^ | 0.60±0.13^B^ | 0.81±0.05^Ab^ | 0.86±0.06^A^ | 0.74±0.13 | 0.90±0.13^ab^ | 0.77±0.22 |
| ƩSFA | 33.94±0.72^Aa^ | 35.28±1.23^A^ | 30.37±1.79^Bb^ | 29.26±1.12^Bb^ | 34.19±1.68^A^ | 36.51±1.62^Aa^ | 35.08±1.01^a^ | 35.32±1.24 | 34.31±1.51^a^ |
| Monounsaturated fatty acids | |  |  |  |  |  |  |  |  |
| C14:1n5 | 0.55±0.26^c^ | 0.74±0.16^c^ | 0.69±0.20^b^ | 1.00±0.25^b^ | 1.00±0.17^b^ | 1.41±0.24^a^ | 1.37±0.16^a^ | 1.28±0.10^a^ | 1.28±0.05^a^ |
| C16:1n5 | 1.12±0.26^b^ | 0.96±0.20^b^ | 0.96±0.19^b^ | 1.30±0.36^b^ | 0.90±0.29^b^ | 1.40±0.62^b^ | 2.22±0.44^a^ | 2.78±0.87^a^ | 2.97±0.63^a^ |
| C17:1n7 | 0.34±0.13 | 0.30±0.06 | 0.26±0.05^b^ | 0.45±0.20^AB^ | 0.25±0.09^B^ | 0.59±0.11^Aa^ | 0.34±0.06 | 0.40±0.11 | 0.37±0.12^b^ |
| C18:1n9 | 2.07±0.16 | 2.22±0.16^b^ | 1.87±0.23^b^ | 2.08±0.14^B^ | 1.89±0.13^Bc^ | 2.57±0.34^Aa^ | 2.25±0.13^B^ | 2.63±0.14^Aa^ | 2.70±0.24^Aa^ |
| C20:1n9 | 0.28±0.07 | 0.33±0.05 | 0.28±0.02 | 0.26±0.04 | 0.26±0.05 | 0.33±0.09 | 0.27±0.09 | 0.33±0.04 | 0.31±0.05 |
| C22:1n9 | 0.07±0.01 | 0.10±0.02 | 0.08±0.03 | 0.07±0.02 | 0.09±0.05 | 0.08±0.03 | 0.10±0.04 | 0.09±0.03 | 0.10±0.02 |
| C24:1n9 | 2.64±0.29 | 2.54±0.39^a^ | 2.34±0.36^ab^ | 2.18±0.15 | 2.06±0.10^b^ | 2.69±0.57^a^ | 2.36±0.35^A^ | 1.95±0.20^Bb^ | 1.73±0.08^Bb^ |
| ƩMUFA | 7.07±0.58^b^ | 7.20±0.49^b^ | 6.46±0.71^b^ | 7.32±0.35^Bb^ | 6.46±0.39^Bb^ | 9.07±1.30^Aa^ | 8.90±0.47^a^ | 9.47±0.63^a^ | 9.46±0.66^a^ |
| Polyunsaturated fatty acids | |  |  |  |  |  |  |  |  |
| C18:2n6 | 10.58±1.13^A^ | 9.43±0.54^Ab^ | 7.43±1.01^Bb^ | 11.7±1.41^B^ | 7.06±1.02^Cc^ | 13.56±0.86^Aa^ | 11.90±0.88^C^ | 13.35±1.04^Ba^ | 14.86±0.48^Aa^ |
| C18:3n3 | 0.12±0.01 | 0.11±0.00 | 0.11±0.02^b^ | 0.1±0.02 | 0.09±0.03 | 0.11±0.02^b^ | 0.09±0.03 | 0.15±0.07 | 0.20±0.06^a^ |
| C18:3n6 | 0.16±0.06^b^ | 0.16±0.04^b^ | 0.15±0.05^b^ | 0.26±0.12^ab^ | 0.13±0.06^b^ | 0.19±0.08^b^ | 0.36±0.11^a^ | 0.58±0.29^a^ | 0.67±0.24^a^ |
| C20:2n6 | 0.80±0.18^A^ | 0.51±0.08^Bb^ | 0.59±0.12^ABb^ | 0.61±0.19 | 0.55±0.09^b^ | 0.76±0.10^a^ | 0.71±0.08 | 0.81±0.17^a^ | 0.82±0.05^a^ |
| C20:3n3 | 0.79±0.03 | 0.74±0.06^b^ | 0.69±0.10 | 0.73±0.11 | 0.69±0.05^b^ | 0.88±0.24 | 0.93±0.20 | 0.98±0.20^a^ | 0.96±0.04 |
| C20:3n6 | 1.32±0.16^Aa^ | 0.89±0.11^B^ | 0.91±0.19^B^ | 0.89±0.21^b^ | 0.84±0.09 | 1.00±0.24 | 0.91±0.05^b^ | 0.90±0.06 | 0.96±0.07 |
| C20:4n6 | 3.28±0.22^a^ | 3.20±0.29 | 3.02±0.27 | 2.68±0.22^b^ | 3.11±0.27 | 2.86±0.16 | 2.99±0.27^ab^ | 2.72±0.18 | 2.76±0.49 |
| C20:5n3 | 2.36±0.37^b^ | 2.06±0.29^b^ | 2.13±0.38^b^ | 2.34±0.36^b^ | 2.14±0.23^b^ | 2.45±0.38^b^ | 3.03±0.27^a^ | 3.19±0.51^a^ | 3.24±0.19^a^ |
| C22:2n6 | 0.37±0.10 | 0.42±0.11 | 0.43±0.10 | 0.40±0.06 | 0.46±0.14 | 0.42±0.08 | 0.38±0.10 | 0.51±0.15 | 0.49±0.05 |
| C22:6n3 | 39.21±1.56^Bb^ | 40.01±1.22^Bb^ | 47.71±1.61^Aa^ | 43.71±1.32^Aa^ | 44.27±0.98^Aa^ | 32.16±1.54^Bb^ | 34.71±0.68^Ac^ | 32.02±1.25^Bc^ | 31.28±1.17^Bb^ |
| ƩPUFA | 58.99±0.77^Bb^ | 57.52±1.00^Bb^ | 63.17±1.79^Aa^ | 63.42±0.91^Aa^ | 59.34±1.45^Ba^ | 54.42±1.81^Bb^ | 56.02±1.12^c^ | 55.21±0.80^c^ | 56.23±1.22^b^ |

Note: Same as Supplementary Table S4.
