# Supplementary material for: Effects of constant and diel cyclic temperatures on the liver and intestinal phospholipid fatty acid composition in rainbow trout Oncorhynchus mykiss during seawater acclimation
Source: BMC Zool. 2021 Jun 21;6:21. doi: 10.1186/s40850-021-00086-6 (PMC10127026; doi:10.1186/s40850-021-00086-6)
Supplement: Supplementary file 3 — Additional file 3 P values of two-way ANOVA for the effects of temperature variations and seawater acclimation on PLFA-related indices in rainbow trout. [file 40850_2021_86_MOESM3_ESM.docx]

**Supplementary Table** **S6** The *P* values of two-way ANOVA for the effects of temperature variations and seawater acclimation on PLFA-related indices in rainbow trout

| PLFA indices | | SFA | MUFA | PUFA | n-3 PUFA | n-6 PUFA | n3/n6 | U/S | UI | ACL |
| --- | --- | --- | --- | --- | --- | --- | --- | --- | --- | --- |
| Constant temperature | | | |  |  |  |  |  |  |  |
| Liver | Model | <.0001 | <.0001 | <.0001 | <.0001 | <.0001 | <.0001 | <.0001 | <.0001 | <.0001 |
|  | Temp. | <.0001 | <.0001 | <.0001 | <.0001 | <.0001 | <.0001 | <.0001 | <.0001 | <.0001 |
|  | Time | <.0001 | <.0001 | 0.0025 | <.0001 | <.0001 | <.0001 | <.0001 | <.0001 | <.0001 |
|  | Interact. | 0.0027 | <.0001 | <.0001 | <.0001 | <.0001 | <.0001 | 0.0009 | <.0001 | <.0001 |
| Intestine | Model | <.0001 | <.0001 | <.0001 | <.0001 | <.0001 | <.0001 | <.0001 | <.0001 | <.0001 |
|  | Temp. | <.0001 | <.0001 | <.0001 | <.0001 | <.0001 | <.0001 | <.0001 | <.0001 | <.0001 |
|  | Time | <.0001 | <.0001 | <.0001 | <.0001 | <.0001 | <.0001 | <.0001 | <.0001 | <.0001 |
|  | Interact. | <.0001 | <.0001 | <.0001 | <.0001 | <.0001 | <.0001 | <.0001 | <.0001 | <.0001 |
|  | |  |  |  |  |  |  |  |  |  |
| Diel cyclic temperature | | | |  |  |  |  |  |  |  |
| Liver | Model | <.0001 | <.0001 | 0.0035 | 0.0017 | 0.0004 | <.0001 | <.0001 | 0.0200 | 0.0058 |
|  | Temp. | 0.8751 | 0.1872 | 0.8565 | 0.3523 | 0.8344 | 0.5296 | 0.8905 | 0.3949 | 0.1522 |
|  | Time | <.0001 | <.0001 | 0.0005 | 0.4637 | 0.0013 | 0.0074 | <.0001 | 0.1874 | 0.1951 |
|  | Interact. | 0.0241 | 0.0684 | 0.0560 | 0.0003 | 0.0009 | <.0001 | 0.0158 | 0.0080 | 0.0027 |
| Intestine | Model | <.0001 | <.0001 | <.0001 | <.0001 | <.0001 | <.0001 | <.0001 | <.0001 | <.0001 |
|  | Temp. | 0.0023 | 0.0610 | 0.0010 | 0.0006 | 0.0002 | 0.0017 | 0.0013 | 0.0008 | 0.0004 |
|  | Time | 0.0078 | <.0001 | <.0001 | <.0001 | <.0001 | <.0001 | 0.0036 | <.0001 | <.0001 |
|  | Interact. | <.0001 | 0.0004 | <.0001 | <.0001 | <.0001 | <.0001 | <.0001 | <.0001 | <.0001 |

Note: Temp: temperature, Interact: interaction, SFA: saturated fatty acid, MUFA: monounsaturated fatty acid, PUFA: polyunsaturated fatty acid, n-3 PUFA: Omega-3 series polyunsaturated fatty acid, n-6 PUFA: Omega-6 series polyunsaturated fatty acid, U/S: the ratio of the unsaturated to saturated fatty acids, UI: unsaturation index, ACL: average chain length.
